# Total Syntheses of Cathepsin D Inhibitory Izenamides A, B, and C and Structural Confirmation of Izenamide B

**DOI:** 10.3390/molecules24193424

**Published:** 2019-09-20

**Authors:** Changjin Lim

**Affiliations:** College of Pharmacy, CHA University, 120 Haeryong-ro, Pocheon 11160, Gyeonggi-do, Korea; koryoi@cha.ac.kr; Tel.: +82-31-881-7193

**Keywords:** izenamides, cathepsin D, depsipeptide, total synthesis, structural confirmation

## Abstract

The first total syntheses of izenamides A, B, and C, which are depsipeptides inhibitor of cathepsin D, were accomplished. In addition, the stereochemistry of izenamide B was confirmed by our syntheses. The key features of our synthetic route involve the avoidance of critical 2,5-diketopiperazine (DKP) formation and the minimization of epimerization during the coupling of amino acids for the target peptides.

## 1. Introduction

Cathepsin D is an aspartic protease and has attracted considerable attention from both biologists and chemists due to its interesting biological functions [1,2]. This aspartic protease, which is involved in protein catabolism and required in certain epithelial cells for tissue remodeling and renewal, regulates lysosomal proteolysis and endogenous fibrinolysis [3,4,5,6]. Cathepsin D also maintains hormone and antigen processing [7,8,9]. In particular, cathepsin D is involved in various diseases, including breast cancer metastasis [10,11,12], atherosclerosis [8,13], Alzheimer’s disease [14,15,16] and neuronal ceroid lipofuscinosis (NCL) [6,17,18,19]. Therefore, therapeutic modulators of the proteolytic activity of cathepsin D have been extensively investigated for the development of new drugs although there have been few reports regarding inhibitors of cathepsin D [20,21,22,23,24].

**Figure 1:**
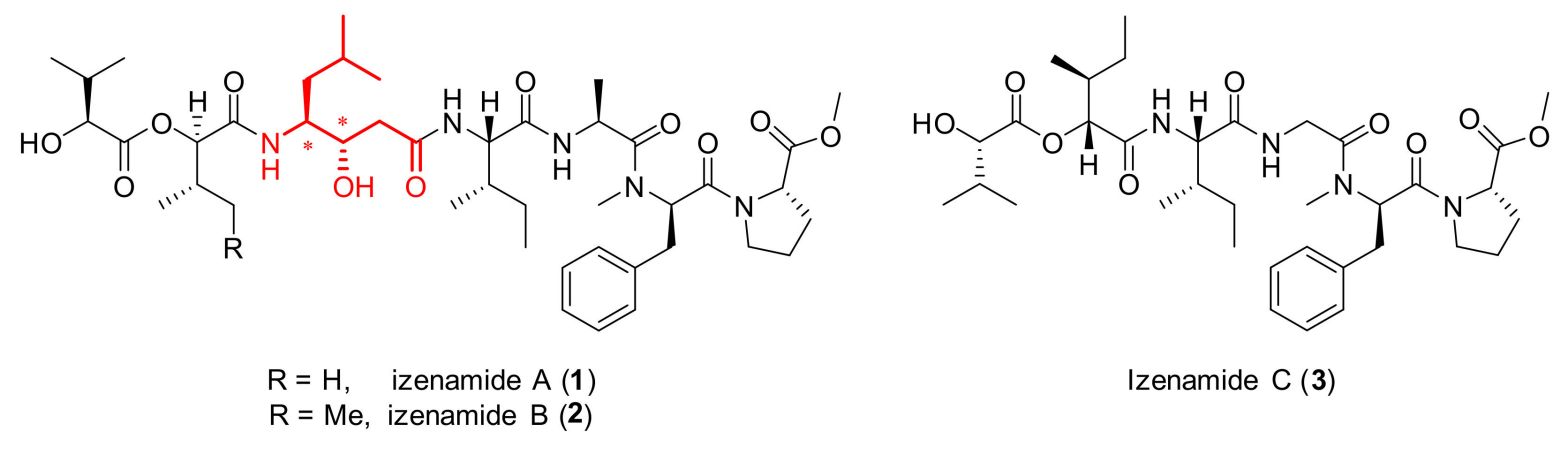
Structures of Izenamides.

Recently, three new depsipeptides, namely, izenamides A (**1**), B (**2**), and C (**3**), were isolated from the marine cyanobacterium 1605-5 by Suenaga and coworkers (Figure 1) [25]. Interestingly, izenamides A (**1**) and B (**2**) were shown to inhibit cathepsin D in vitro. These two depsipeptides consist of seven monomers, including four amino acids, two hydroxy acids, and a γ-amino-β-hydroxy acid called statine. Izenamide C (**3**), which lacks a statine moiety and possesses a glycine unit instead of the alanine seen in izenamides A (**1**) and B (**2**), has no inhibitory activity against cathepsin D. The absolute configuration of the statine moiety in izenamide B (**2**) was indirectly determined by numerous efforts by Suenaga and coworkers [25].

In our pursuit of developing novel cathepsin D inhibitors, we recently became interested in establishing an efficient synthetic route towards izenamides A, B, and C. We were also interested in confirming the structure of izenamide B to ensure the stereochemistry of the statine moiety before we started medicinal chemistry studies. We herein report the first total syntheses of izenamides A, B, and C as well as the structural confirmation of izenamide B.

## 2. Results and Discussion

### 2.1. Synthetic Strategy for Izenamides A (**1**), B (**2**), and C (**3**)

**Scheme 1:**
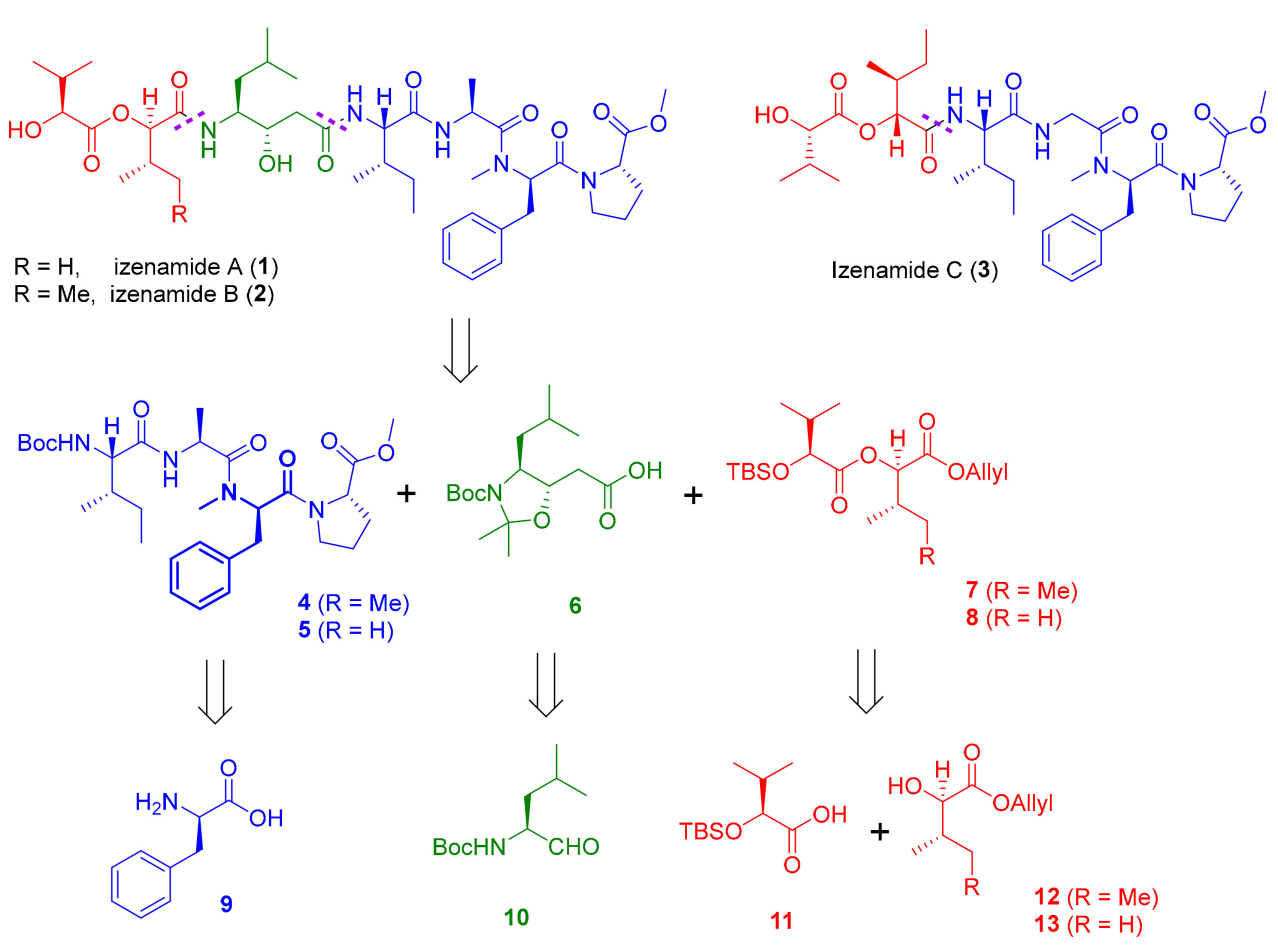
Retrosynthetic analysis for the synthesis of izenamides **1, 2** and **3.**

Our retrosynthetic analysis is outlined in Scheme 1. We envisioned that the target izenamides could be accessed via a versatile convergent approach. Izenamides A (**1**) and B (**2**) would be synthesized by assembling three fragments, tetrapeptide **4**, statine **6**, and esters **7** or **8**. Considering that izenamide C (**3**) has a very similar structure except for the statine moiety, it could also be convergently synthesized by the amide coupling of tetrapeptide **5** and ester **7**. Tetrapeptides **4** and **5** were anticipated to be derived from commercially available amino acids and *N*Me-d-Phe, which could be obtained from d-Phe (**9**). Protected statine **6** could be prepared from Boc-l-leucinal (**10**) through the known procedure [26,27]. Esters **7** and **8** can be conveniently obtained by the esterification of protected hydroxy acids **11** and **12** (or **13**).

### 2.2. Syntheses of Fragments

**Scheme 2:**
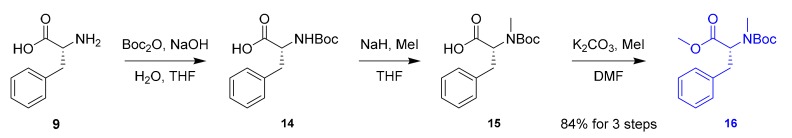
Preparation of NMe-d-Phe monomer **16.**

The synthesis of izenamides commenced with synthesizing tetrapeptides **4** and **5**. Initially, *N*Me-d-Phe **16** was prepared from commercially available d-Phe (**9**) as shown in Scheme 2. Amine protection of d-Phe **9** with Boc anhydride and *N*-methylation of resulting carbamate **14** afforded acid **15**, which was esterified to produce desired monomer **16** in 84% yield over three steps.

**Scheme 3:**
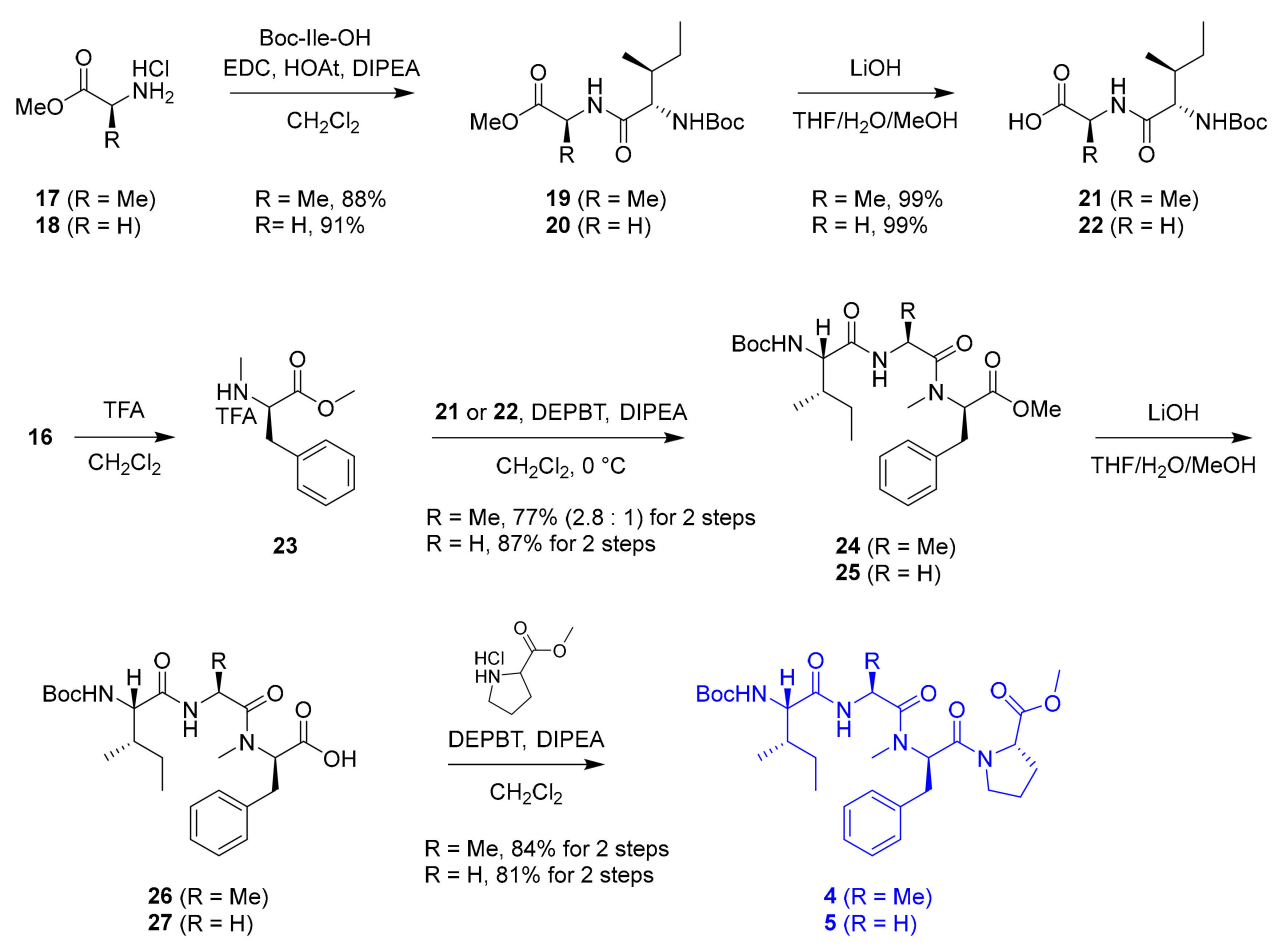
Synthesis of tetrapeptides **4** and **5.**

With monomer **16** in hand, we turned our attention to the synthesis of tetrapeptides **4** and **5** (Scheme 3). To avoid facile DKP formation upon sequential elongation from the *C*-terminal *N*Me-Phe and Pro residue, we synthesized tetrapeptides **4** and **5** according to the optimized sequence of fragment couplings. Amidation of l-alanine methyl ester hydrochloride (**17**) and glycine methyl ester hydrochloride (**18**) with Boc-Ile-OH followed by ester hydrolysis with LiOH afforded dipeptides **21** and **22** in high yields. Deprotection of **16** and coupling of resulting free amine **23** with acids **21** and **22** produced tripeptides **24** and **25** in 77 and 87% yields over two steps, respectively. Unfortunately, epimerization was observed during the amidation to form **24**. After thorough optimization of the coupling conditions, amidation in the presence of DEPBT in CH_2_Cl_2_ at 0 °C provided the best results in terms of high chemical yield and minimal epimerization (2.8:1). Tripeptides **24** and **25** were subjected to hydrolysis and then amidation with l-proline methyl ester hydrochloride to afford tetrapeptides **4** and **5** in 84 and 81% yield over two steps, respectively. In general, the *N*-Me peptide backbone near the *C*-terminal is prone to α-epimerization upon carboxy activation due to steric hindrance and electronic effects [28]. However, we could obtain the desired tetrapeptides without epimerization under the optimized conditions (DEPBT, CH_2_Cl_2_, 0 °C).

**Scheme 4:**
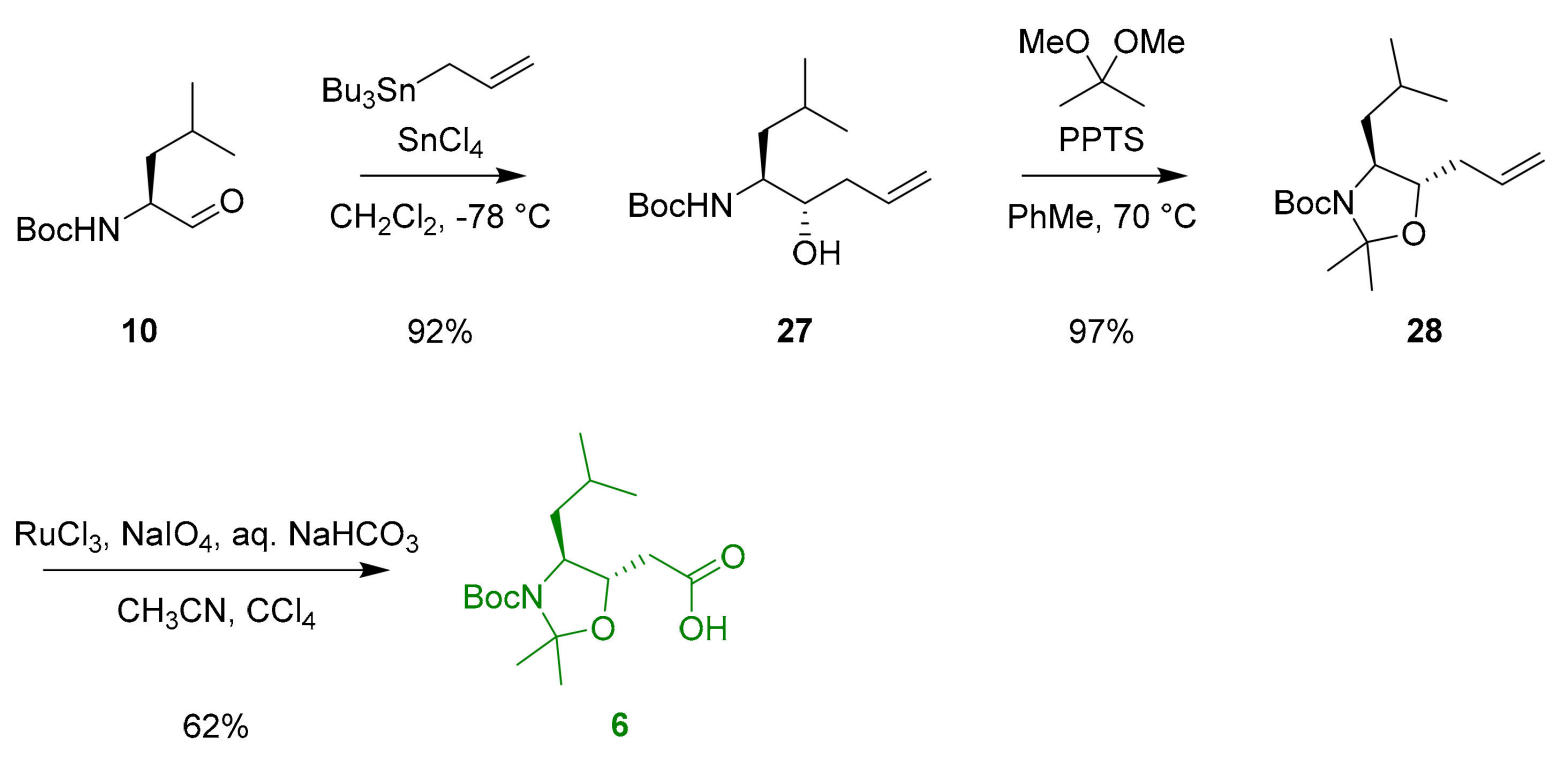
Synthesis of statine **6.**

Optically pure statine **6** was prepared by the known procedure [26,27] (Scheme 4). An elegant highly diastereoselective allyl addition to *N*-Boc-l-leucinal in the presence of SnCl_4_ [26] provided desired threo isomer **27**. The reaction of homoallylic alcohol **27** with 2,2-dimethoxypropane in the presence of catalytic PPTS followed by RuO_4_-mediated oxidative degradation [27] afforded monomer acid **6**.

**Scheme 5:**
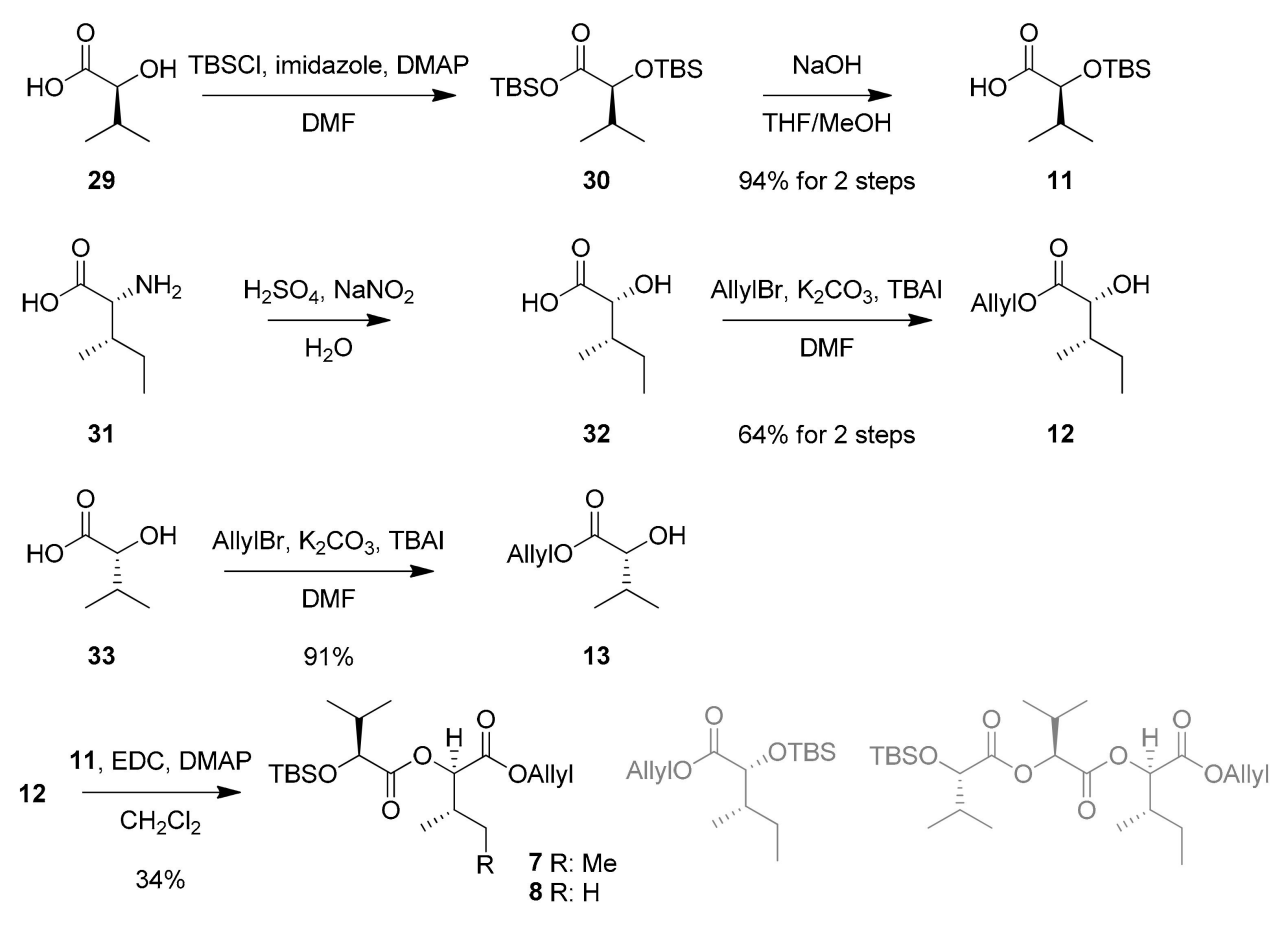
Synthesis of Ester **7.**

The synthesis of esters **7** and **8,** which are the C-terminal fragments of izenamides, began with the preparation of silyl-protected valic acid **11** (Scheme 5). Global TBS-protection of commercially available l-valic acid (**29**) and hydrolysis of resulting silyl ester **30** produced *C*-terminal acid **11** in 94% yield over 2 steps. d-Allo-isoleucic acid **32 [29]**, which was prepared through the diazotization of commercially available d-allo-Ile (**31**), and d-valic acid (**33**) were converted to corresponding allyl esters **12** (64% for 2 steps) and **13** (91%), respectively. The EDC-mediated esterification of acid **11** with alcohol **12** afforded ester **7**. Unfortunately, a satisfactory yield for this esterification was not achieved due the production of undesired esters by inevitable silyl transfer between hydroxyl groups.

**Scheme 6:**
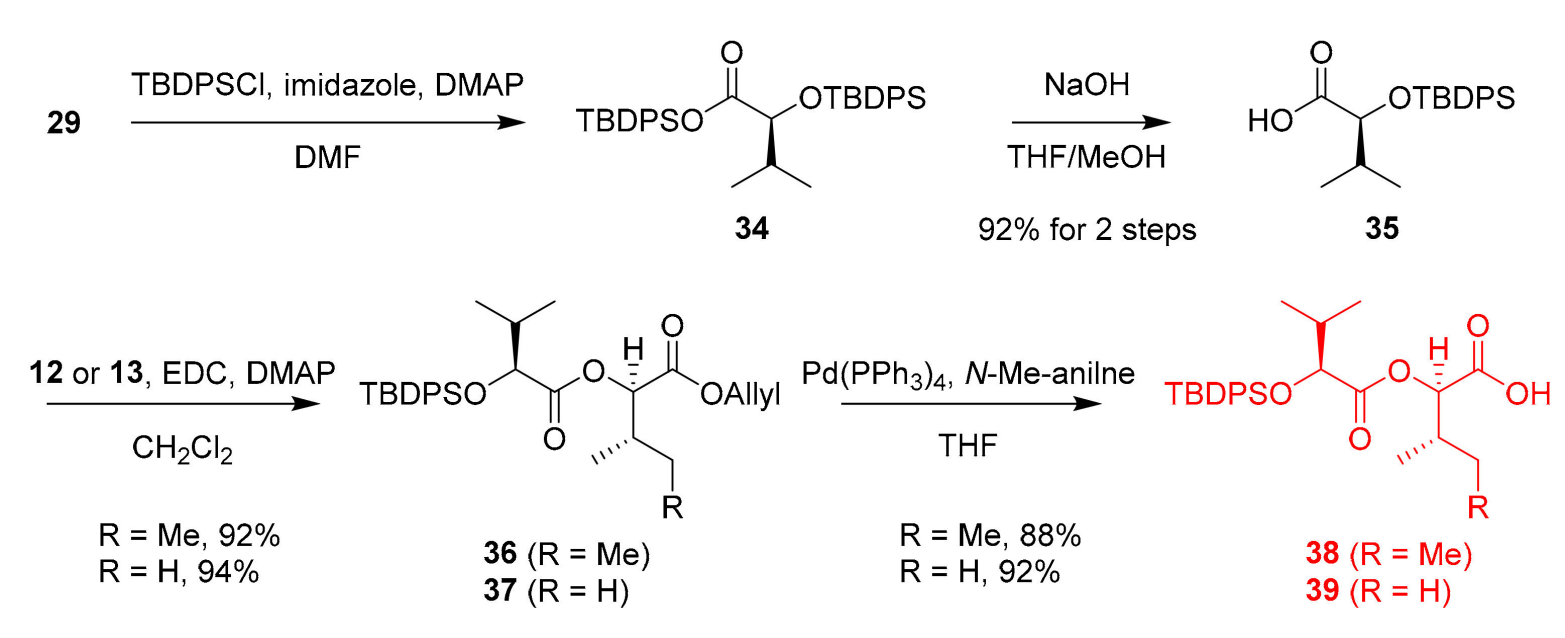
Synthesis of fragments **38** and **39** with minimized silyl transfer.

Therefore, we decided to change the TBS protecting group to a bulkier TBDPS group, which was anticipated to minimize silyl transfer (Scheme 6). To our delight, the silyl transfer was minimized in the coupling reactions, and fragments **36** and **37** were obtained in 92% and 94% yields, respectively. Finally, allyl deprotection of esters **36** and **37** with *N*-Me-aniline in the presence of Pd(0) produced acids **38** in 88% and **39** in 92% yield, respectively.

### 2.3. Completion of the Syntheses

**Scheme 7:**
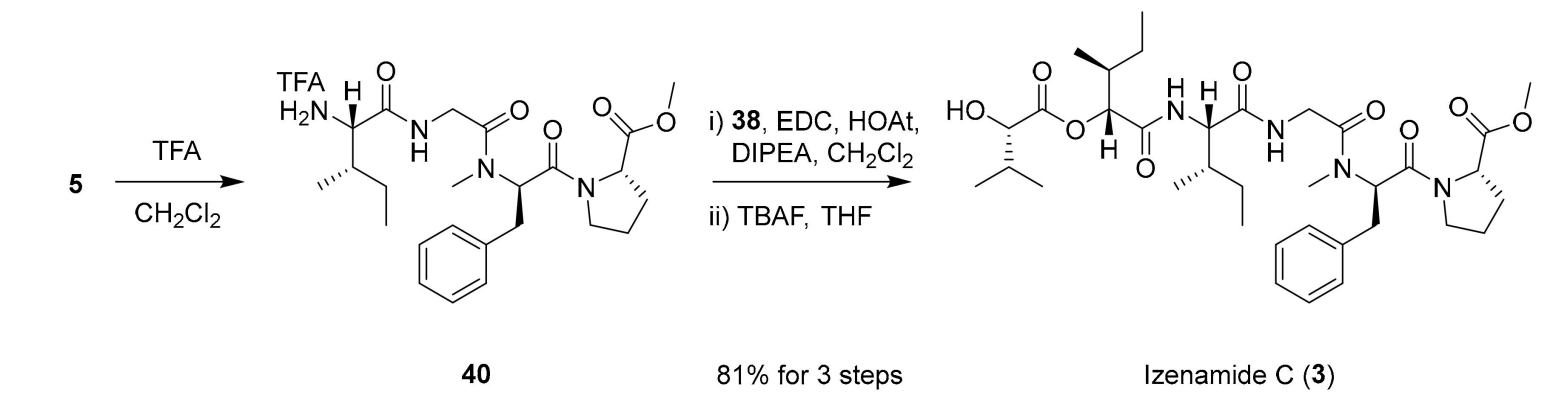
Completion of the synthesis of izenamide C (**3**).

We finally assembled the prepared fragments of izenamide C as shown in Scheme 7. The EDC-mediated amide coupling of amine **40**, which was prepared from tetrapeptide **5,** with acid **38** followed by silyl-deprotection produced izenamide C (**3**) in 81% over 3 steps.

**Scheme 8:**
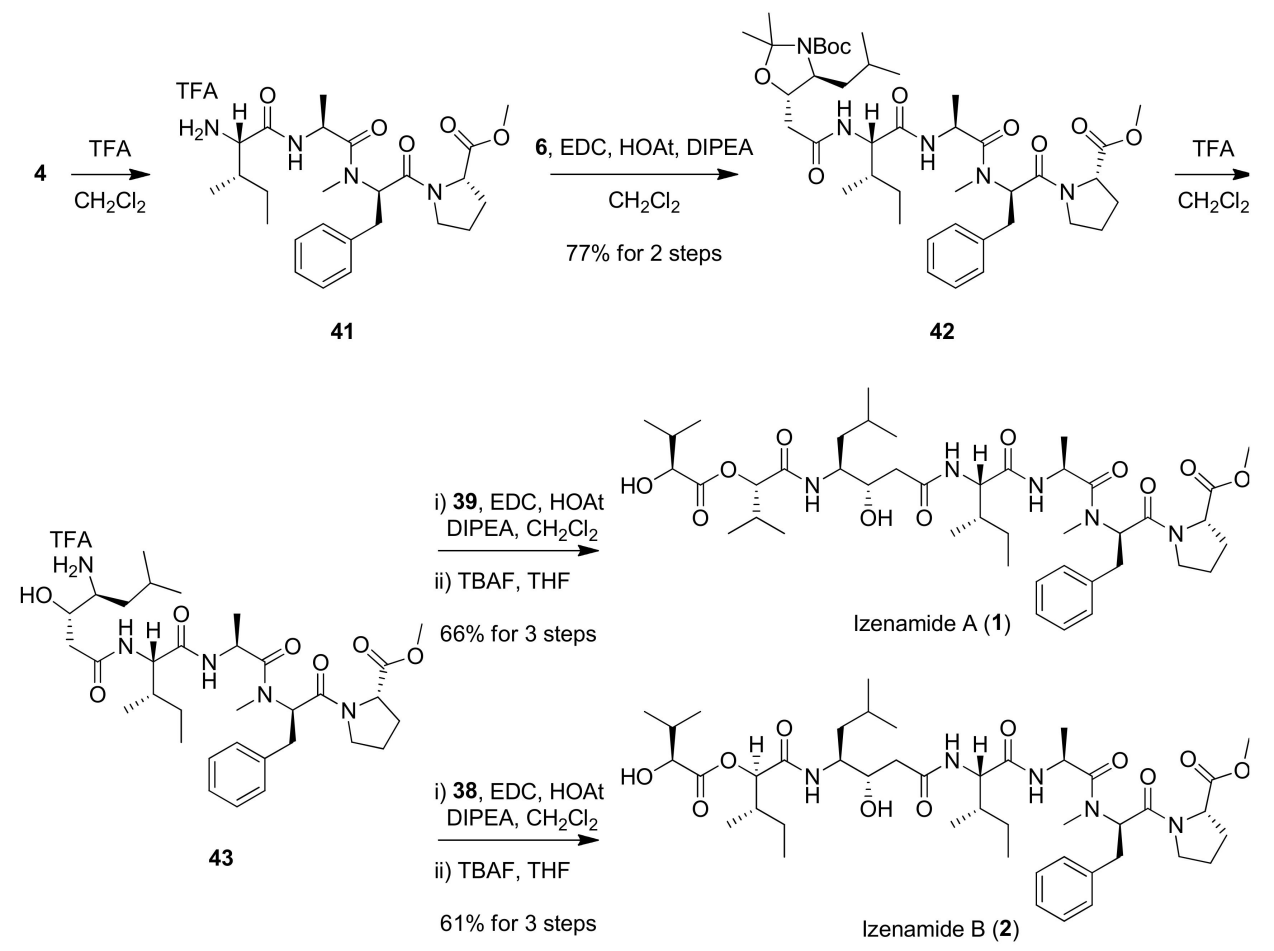
Completion of the syntheses of izenamides A (**1**) and B (**2**).

The completed synthesis of izenamides A (**1**) and B (**2**) is illustrated in Scheme 8. Boc-deprotection of tetrapeptide **4** with TFA and amide coupling with acid **6** afforded pentapeptide **42** in 77% yield over two steps. Global deprotection under acidic conditions followed by amidation of the resulting amine with acids **39** or **38** produced the corresponding heptapeptides. Finally, desilylation of heptapeptides afforded izenamides A (**1**) and B (**2**) in 66% and 61% over 3 steps, respectively. Spectral data of the synthesized depsipeptides **1**, **2**, and **3** were all identical with the reported data of natural **1**, **2** and **3 [25]**. Moreover, the absolute configuration of the statine moiety in **2** was identical to that of **1**. The ^1^H and ^13^C NMR spectra of some compounds are in the Supplementary Materials.

## 3. Materials and Methods

### 3.1. General Information

Unless noted otherwise, all starting materials and reagents were obtained from commercial suppliers (Sigma-Aldrich, St. Louis, MO, USA; TCI, Tokyo, Japan; Combi-Blocks, San Diego, CA, USA) and were used without further purification. Tetrahydrofuran and Et_2_O were distilled from sodium benzophenone ketyl. Dichloromethane, chloroform and acetonitrile were freshly distilled from calcium hydride. All solvents used for routine isolation of products and chromatography were reagent grade and glass distilled. Reaction flasks were dried at 100 °C. Air and moisture sensitive reactions were performed under argon atmosphere. Flash column chromatography was performed using silica gel 60 (230–400 mesh, Merck, Kenilworth, NJ, USA) with the indicated solvents. Thin-layer chromatography was performed using 0.25 mm silica gel plates (Merck, Kenilworth, NJ, USA). Optical rotations were measured with JASCO P-2000 digital polarimeter (Tokyo, Japan) at ambient temperature using cylindrical cell of 10 mm or 100 mm pathlength. Infrared spectra were recorded on a JASCO FT-IR-4200 spectrometer (Tokyo, Japan). High resolution mass spectra were obtained with JEOL JMS-700 (Tokyo, Japan) and Agilent Q TOF 6530 (Santa Clara, CA, USA) instruments. ^1^H and ^13^C NMR spectra were recorded using BRUKER AVANCE-800 (Billerica, MA, USA). Chemical shifts are expressed in parts per million (ppm, δ) downfield from tetramethylsilane and are referenced to the deuterated solvent (CHCl_3_, ^1^H δ 7.24, ^13^C δ 77.0; MeOH-*d_4_*, ^1^H δ 3.30, ^13^C δ 49.00). ^1^H*-*NMR data were reported in the order of chemical shift, multiplicity (s, singlet; d, doublet; t, triplet; q, quartet; m, multiplet and/or multiple resonances; br, broad signal), coupling constant in hertz (Hz) and number of protons.

### 3.2. Experimental Part

*Boc-NMe-d-Phe-OMe* (**16**). To a solution of d-Phe **9** (1.7 g, 10.3 mmol) and Boc_2_O (3.5 mL, 15.4 mmol) in a mixture THF and H_2_O (1:1, 50 mL) was added NaOH (0.6 g, 15.4 mmol) at room temperature. After stirring overnight, the reaction mixture was quenched with 1*N* HCl and extracted with EtOAc. The combined organic layer was washed with brine, dried over MgSO_4_, and concentrated *in vacuo*. To a solution of above crude Boc-d-Phe-OH **14** (10.3 mmol) in dry THF (20 mL) was added NaH (60% dispersion in mineral oil, 2.1 g, 51.5 mmol) at room temperature. After stirring for 1 h, iodomethane (3.2 mL, 51.5 mmol) was added to the reaction mixture. The reaction mixture was stirred for 12 h, quenched with 1*N* HCl, and extracted with EtOAc. The combined organic layer was washed with brine, dried over MgSO_4_, and concentrated *in vacuo*. The residue was used in the next step without further purification. To a solution of crude acid **15** (10.3 mmol) in dry DMF (20 mL) were added iodomethane (1.3 mL, 20.6 mmol) and K_2_CO_3_ (2.8 g, 20.6 mmol) at room temperature. After stirring overnight, the reaction mixture was quenched with 1*N* HCl and extracted with Et_2_O. The combined organic layer was washed with brine, dried over MgSO_4_, and concentrated *in vacuo*. The residue was purified by flash column chromatography (EtOAc/Hexane = 1:20) to give 2.5 g (84% for 3 steps) of ester **16** as a colorless oil. [α]_D_^20^ = +109.84 (*c* 1.00, CHCl_3_); ^1^H-NMR (800 MHz, CDCl_3_, 3:2 mixture of two rotamers). Major rotamer δ 7.28–7.20 (m, 2H), 7.19–7.07 (m, 3H), 4.50 (dd, *J* = 10.4, 3.8 Hz, 1H), 3.70 (s, 3H), 3.23 (dd, *J* = 14.2, 4.4 Hz, 1H), 3.01–2.94 (m, 1H), 2.68 (s, 3H), 1.29 (s, 9H), minor rotamer δ 7.28–7.20 (m, 2H), 7.19–7.07 (m, 3H), 4.89 (dd, *J* = 10.6, 5.2 Hz, 1H), 3.68 (s, 3H), 3.27 (dd, *J* = 14.4, 5.1 Hz, 1H), 3.01–2.94 (m, 1H), 2.66 (s, 3H), 1.33 (s, 9H); ^13^C-NMR (200 MHz, CDCl_3_, 3:2 mixture of two rotamers). Major rotamer δ 171.4, 154.8, 137.5, 128.9, 128.4, 126.5, 80.1, 61.5, 52.0, 35.4, 32.4, 28.0, minor rotamer δ 171.7, 155.6, 137.2, 128.8, 128.2, 126.3, 79.8, 59.4, 52.0, 34.9, 31.8, 28.1; IR (thin film, neat) *ν*_max_ 2977, 1746, 1698, 1393, 1332, 1227, 1145, 751 cm^−1^; LR-MS (ESI+) *m/z* 316 (M + Na^+^); HR-MS (ESI+) calcd for C_16_H_23_NNaO_4_ (M + Na^+^) 316.1519; found 316.1523.

*Boc-L-Ile-L-Ala-OH* (**21**). To a solution of l-alanine methyl ester hydrochloride **17** (1.7 g, 12.2 mmol), Boc-l-Ile-OH (2.3 g, 9.9 mmol), DIPEA (5.2 mL, 29.8 mmol), and HOAt (1.4 g, 10.4 mmol) in CH_2_Cl_2_ (40 mL) was added EDC∙HCl (3.8 g, 19.9 mmol) at room temperature. After stirring for 4 h, the reaction mixture was quenched with 1*N* HCl, extracted with CH_2_Cl_2_. The combined organic layer was washed with aqueous NaHCO_3_, dried over MgSO_4_, and concentrated *in vacuo*. The residue was purified by flash column chromatography (EtOAc/Hexane = 1:2 to 1:1) to give 2.8 g (88%) of dipeptide **19** as white solid. [α]_D_^20^ = +16.54 (*c* 1.00, CHCl_3_); ^1^H NMR (800 MHz, CDCl_3_) δ 6.40 (s, 1H), 5.03 (d, *J* = 6.7 Hz, 1H), 4.56 (dq, *J* = 7.2, 7.1 Hz, 1H), 3.94 (t, *J* = 6.2 Hz, 1H), 3.72 (s, 3H), 1.88–1.81 (m, 1H), 1.53–1.44 (m, 1H), 1.42 (s, 9H), 1.39 (d, *J* = 7.2 Hz, 3H), 1.17–1.09 (m, 1H), 0.92 (d, *J* = 6.8 Hz, 3H), 0.89 (t, *J* = 7.4 Hz, 3H); ^13^C NMR (200 MHz, CDCl_3_) δ 173.1, 171.1, 155.7, 79.9, 59.1, 52.4, 48.0, 37.3, 28.3, 24.7, 18.3, 15.4, 11.4; IR (thin film, neat) *ν*_max_ 3313, 2977, 1751, 1682, 1651, 1528, 1367, 1252, 870 cm^−1^; LR-MS (ESI+) *m/z* 317 (M + H^+^); HR-MS (ESI+) calcd for C_15_H_29_N_2_O_5_ (M + H^+^) 317.2071; found 317.2072. To a solution of Boc-l**-**Ile*-*l**-**Ala-OMe **19** (2.0 g, 6.3 mmol) in a mixture of THF, MeOH and H_2_O (1:1:1, 10 mL) was added LiOH∙H_2_O (0.6 g, 14.3 mmol) at room temperature. After stirring for 2 h, the reaction mixture was quenched with 1*N* HCl and extracted with EtOAc. The combined organic layer was dried over MgSO_4_ and concentrated *in vacuo* to afford 1.9 g (99%) of Boc-l-Ile-l-Ala-OH **21** as a white solid. The free acid **21** was used in the next step without further purification.

*Boc-L-Ile-Gly-OH* (**22**). To a solution of glycine methyl ester hydrochloride **18** (1.5 g, 11.9 mmol), Boc-l-Ile-OH (2.3 g, 9.9 mmol), DIPEA (5.2 mL, 29.8 mmol), and HOAt (1.4 g, 10.4 mmol) in CH_2_Cl_2_ (40 mL) was added EDC∙HCl (3.8 g, 19.9 mmol) at room temperature. After stirring for 5 h, the reaction mixture was quenched with 1*N* HCl and extracted with CH_2_Cl_2_. The combined organic layer was washed with aqueous NaHCO_3_, dried over MgSO_4_, and concentrated *in vacuo*. The residue was purified by flash column chromatography (EtOAc/Hexane = 1:2 to 1:1) to give 2.7 g (91%) of dipeptide **20** as a white solid. [α]_D_^20^ = −5.85 (*c* 1.00, CHCl_3_); ^1^H-NMR (800 MHz, CDCl_3_) δ 6.81 (s, 1H), 5.17 (d, *J* = 7.5 Hz, 1H), 4.08–3.91 (m, 3H), 3.70 (s, 3H), 1.84 (s, 1H), 1.51–1.45 (m, 1H), 1.39 (s, 9H), 1.13–1.06 (m, 1H), 0.91 (d, *J* = 6.9 Hz, 3H), 0.86 (t, *J* = 7.4 Hz, 3H); ^13^C-NMR (200 MHz, CDCl_3_) δ 172.1, 170.1, 155.8, 79.9, 59.0, 52.2, 41.0, 37.2, 28.2, 24.6, 15.4, 11.3; IR (thin film, neat) *ν*_max_ 3324, 2968, 1759, 1687, 1661, 1527, 1367, 1173, 862 cm^−1^; LR-MS (ESI+) *m/z* 303 (M + H^+^); HR-MS (ESI+) calcd for C_14_H_27_N_2_O_5_ (M + H^+^) 303.1914; found 303.1923. To a solution of Boc-l**-**Ile*-*Gly-OMe **20** (2.0 g, 6.6 mmol) in a mixture of THF, MeOH, and H_2_O (1:1:1, 33 mL) was added LiOH∙H_2_O (0.6 g, 14.3 mmol) at room temperature. After stirring for 2 h, the reaction mixture was quenched with 1*N* HCl and extracted with EtOAc. The combined organic layer was dried over MgSO_4_ and concentrated *in vacuo* to afford 1.9 g (99%) of Boc-l-Ile-Gly-OH **22** as white solid. The free acid **22** was used in the next step without further purification.

*Boc-l**-**Ile-l-Ala-NMe-d**-**Phe-OMe* (**24**). To a solution of Boc*-N*Me-d**-**Phe-OMe **16** (440 mg, 1.5 mmol) in CH_2_Cl_2_ (6.0 mL) was added TFA (1.5 mL) dropwise at room temperature. After stirring for 1 h, the reaction mixture was concentrated *in vacuo*. The residue was used in the next step without further purification. To a solution of above amine salt **23** (1.5 mmol), Boc-l-Ile-l-Ala-OH **21** (544 mg, 1.8 mmol), and DIPEA (0.8 mL, 4.6 mmol) in CH_2_Cl_2_ (7.5 mL) was added DEPBT (898 mg, 3.0 mmol) at 0 °C. After stirring for overnight at 0 °C, the reaction mixture was quenched with 1*N* HCl, extracted with CH_2_Cl_2_. The combined organic layer was washed with aqueous NaHCO_3_, dried over MgSO_4_, and concentrated *in vacuo*. The residue was purified by flash column chromatography (EtOAc/Hexane = 1:1) to give 407 mg (57% for 2 steps) of tripeptide **24** as white solid and 145 mg (20% for 2 steps) of its epimer as white solid. [α]_D_^20^ = +61.58 (*c* 1.00, CHCl_3_); ^1^H-NMR (800 MHz, CDCl_3_, a mixture of rotamers) Major rotamer δ 7.24 (t, *J* = 7.4 Hz, 2H), 7.18 (t, *J* = 7.4 Hz, 1H), 7.13 (d, *J* = 7.3 Hz, 2H), 6.81 (d, *J* = 7.1 Hz, 1H), 5.29 (d, *J* = 7.1 Hz, 1H), 4.98 (d, *J* = 8.3 Hz, 1H), 4.69 (dq, *J* = 6.9, 6.8 Hz, 1H ), 3.95 (dd, *J* = 7.3, 6.1 Hz, 1H), 3.73 (s, 3H), 3.39 (dd, *J* = 14.8, 5.0 Hz, 1H), 3.01 (dd, *J* = 14.7, 11.8 Hz, 1H), 2.83 (s, 3H), 1.87–1.79 (m, 1H), 1.74–1.64 (m, 1H), 1.40 (s, 9H), 1.12–1.02 (m, 1H), 0.88 (d, *J* = 6.8 Hz, 3H), 0.87 (t, *J* = 7.4 Hz, 3H), 0.84 (d, *J* = 6.8 Hz, 3H).; ^13^C-NMR (200 MHz, CDCl_3_, a mixture of rotamers) Major rotamer δ 172.9, 170.7, 170.5, 155.6, 136.4, 128.7, 128.6, 126.9, 79.8, 59.2, 58.3, 52.4, 45.5, 37.6, 34.7, 32.5, 28.3, 24.6, 17.9, 15.6, 11.5; IR (thin film, neat) *ν*_max_ 3319, 2970, 1743, 1710, 1637, 1498, 1366, 1173, 1017, 871 cm^−1^; LR-MS (ESI+) *m/z* 478 (M + H^+^); HR-MS (ESI+) calcd for C_25_H_40_N_3_O_6_ (M + H^+^) 478.2912; found 478.2915.

*Boc-l**-**Ile-Gly-NMe-d**-**Phe-OMe* (**25**). To a solution of Boc*-N*Me-d**-**Phe-OMe **16** (426 mg, 1.5 mmol) in CH_2_Cl_2_ (6.0 mL) was added TFA (1.5 mL) dropwise at room temperature. After stirring for 1 h, the reaction mixture was concentrated *in vacuo*. The residue was used in the next step without further purification. To a solution of above amine salt **23** (1.5 mmol), Boc-l-Ile-Gly-OH **22** (502 mg, 1.7 mmol) and DIPEA (0.8 mL, 4.4 mmol) in CH_2_Cl_2_ (7.3 mL) was added DEPBT (869 mg, 2.9 mmol) at 0 °C. After stirring overnight at 0 °C, the reaction mixture was quenched with 1*N* HCl and extracted with CH_2_Cl_2_. The combined organic layer was washed with aqueous NaHCO_3_, dried over MgSO_4_, and concentrated *in vacuo*. The residue was purified by flash column chromatography (EtOAc/Hexane = 1:1) to give 586 mg (87% for 2 steps) of tripeptide **25** as white solid. [α]_D_^20^ = +38.96 (*c* 1.00, CHCl_3_); ^1^H-NMR (800 MHz, CDCl_3_, a mixture of rotamers) Major rotamer δ 7.21 (t, *J* = 7.5 Hz, 2H), 7.15 (t, *J* = 7.4 Hz, 1H), 7.09 (d, *J* = 7.3 Hz, 2H), 6.84 (s, 1H), 5.19 (dd, *J* = 10.7, 5.3 Hz, 1H), 5.10 (d, *J* = 8.4 Hz, 1H), 4.04–4.01 (m, 1H), 3.95 (dd, *J* = 17.9, 3.7 Hz, 1H), 3.81 (dd, *J* = 17.7, 3.1 Hz, 1H), 3.67 (s, 3H), 3.32 (dd, *J* = 14.6, 5.4 Hz, 1H), 2.98 (dd, *J* = 14.6, 10.9 Hz, 1H), 2.74 (s, 3H), 1.81 (s, 1H), 1.44–1.36 (m, 1H), 1.37 (s, 9H), 1.07–1.02 (m, 1H), 0.85 (d, *J* = 6.9 Hz, 3H), 0.83 (t, *J* = 7.4 Hz, 3H); ^13^C-NMR (200 MHz, CDCl_3_, a mixture of rotamers) Major rotamer δ 171.3, 170.6, 168.4, 155.5, 136.4, 128.5, 128.5, 126.8, 79.5, 58.9, 58.7, 52.3, 41.2, 37.5, 34.4, 31.7, 28.1, 24.5, 15.4, 11.4; IR (thin film, neat) *ν*_max_ 3328, 2967, 1743, 1712, 1643, 1497, 1366, 1172, 1016, 867 cm^−1^; LR-MS (ESI+) *m/z* 464 (M + H^+^); HR-MS (ESI+) calcd for C_24_H_38_N_3_O_6_ (M + H^+^) 464.2755; found 464.2751.

*Boc-l**-**Ile-l-Ala-NMe-d**-**Phe-l**-**Pro-OMe* (**4**). To a solution of Boc-l**-**Ile*-*l**-**Ala*-N*Me-d**-**Phe-OMe **24** (491 mg, 1.0 mmol) in a mixture of THF, MeOH, and H_2_O (1:1:1, 10 mL) was added LiOH∙H_2_O (86 mg, 2.1 mmol) at room temperature. After stirring for 2 h, the reaction mixture was quenched with 1*N* HCl and extracted with EtOAc. The combined organic layer was dried over MgSO_4_ and concentrated *in vacuo*. The free acid **26** was used in the next step without further purification. To a solution of above acid **26** (1.0 mmol), l-proline methyl ester hydrochloride (203 mg, 1.3 mmol), and DIPEA (0.6 mL, 3.4 mmol) in CH_2_Cl_2_ (10 mL) was added DEPBT (615 mg, 2.1 mmol) at 0 °C. After stirring for overnight at 0 °C, the reaction mixture was quenched with 1*N* HCl and extracted with CH_2_Cl_2_. The combined organic layer was washed with aqueous NaHCO_3_, dried over MgSO_4_, and concentrated *in vacuo*. The residue was purified by flash column chromatography (EtOAc/Hexane = 1:1 to 2:1) to give 496 mg (84% for 2 steps) of tetrapeptide **4** as white solid. [α]_D_^20^ = +45.90 (*c* 1.00, CHCl_3_); ^1^H-NMR (800 MHz, CDCl_3_, a mixture of rotamers) Major rotamer δ 7.23–7.13 (m, 5H), 6.64 (d, *J* = 7.2 Hz, 1H), 5.66 (dd, *J* = 9.5, 6.4 Hz, 1H), 4.98 (d, *J* = 8.2 Hz, 1H), 4.74 (dq, *J* = 7.1, 6.9 Hz, 1H), 4.42 (t, *J* = 7.5 Hz, 1H), 3.90 (t, *J* = 6.4 Hz, 1H), 3.70 (s, 3H), 3.47–3.42 (m, 1H), 3.22–3.15 (m, 1H), 2.98 (s, 3H), 2.94 (dd, *J* = 14.4, 9.6 Hz, 1H), 2.22–2.17 (m, 1H), 1.97–1.76 (m, 6H), 1.39 (s, 9H), 1.08–1.01 (m, 1H), 0.84 (t, *J* = 7.4 Hz, 3H), 0.83 (d, *J* = 6.8 Hz, 3H), 0.82 (d, *J* = 6.9 Hz, 3H); ^13^C-NMR (200 MHz, CDCl_3_, a mixture of rotamers) Major rotamer δ 172.3, 172.2, 170.5, 168.1, 155.5, 136.7, 129.5, 128.2, 126.6, 79.7, 59.2, 59.0, 55.5, 52.2, 46.9, 45.1, 37.6, 34.6, 30.2, 28.8, 28.2, 25.3, 24.6, 17.7, 15.4, 11.5; IR (thin film, neat) *ν*_max_ 3316, 2971, 1748, 1709, 1643, 1497, 1365, 1245, 1174, 1045, 870 cm^−1^; LR-MS (ESI+) *m/z* 575 (M + H^+^); HR-MS (ESI+) calcd for C_30_H_47_N_4_O_7_ (M + H^+^) 575.3439; found 575.3442.

*Boc-l**-**Ile-Gly-NMe-d**-**Phe-l**-**Pro-OMe* (**5**). To a solution of Boc-l**-**Ile*-*Gly*-N*Me-d**-**Phe-OMe **25** (511 mg, 1.0 mmol) in a mixture of THF, MeOH, and H_2_O (1:1:1, 11 mL) was added LiOH∙H_2_O (92 mg, 2.2 mmol) at room temperature. After stirring for 2 h, the reaction mixture was quenched with 1*N* HCl and extracted with EtOAc. The combined organic layer was dried over MgSO_4_ and concentrated *in vacuo*. The free acid **27** was used in the next step without further purification. To a solution of above acid **27** (1.1 mmol), l-proline methyl ester hydrochloride (216 mg, 1.4 mmol) and DIPEA (0.6 mL, 3.4 mmol) in CH_2_Cl_2_ (11 mL) was added DEPBT (660 mg, 2.2 mmol) at 0 °C. After stirring overnight at 0 °C, the reaction mixture was quenched with 1*N* HCl, extracted with CH_2_Cl_2_. The combined organic layer was washed with aqueous NaHCO_3_, dried over MgSO_4_, and concentrated *in vacuo*. The residue was purified by flash column chromatography (EtOAc/Hexane = 1:1 to 2:1) to give 501 mg (81% for 2 steps) of tetrapeptide **5** as a white solid. [α]_D_^20^ = +69.20 (*c* 1.00, CHCl_3_); ^1^H-NMR (800 MHz, CDCl_3_, a mixture of rotamers) Major rotamer δ 7.23 (t, *J* = 7.4 Hz, 2H), 7.20 (d, *J* = 6.9 Hz, 2H), 7.17 (t, *J* = 7.2 Hz, 1H), 6.77 (s, 1H), 5.56 (t, *J* = 7.6 Hz, 1H), 5.00 (d, *J* = 8.0 Hz, 1H), 4.40 (dd, *J* = 8.5, 5.7 Hz, 1H), 4.09–4.01 (m, 1H), 3.92–3.85 (m, 1H), 3.70 (s, 3H), 3.38–3.34 (m, 1H), 3.33–3.29 (m, 1H), 3.25 (dd, *J* = 13.7, 8.0 Hz, 1H), 2.96 (s, *J* = 6.5 Hz, 3H), 2.81 (dd, *J* = 13.7, 7.1 Hz, 1H), 2.16–2.11 (m, 1H), 1.95–1.74 (m, 6H), 1.42 (s, 9H), 1.12–1.02 (m, 1H), 0.90 (d, *J* = 6.8 Hz, 3H), 0.88 (t, *J* = 7.4 Hz, 3H).; ^13^C-NMR (200 MHz, CDCl_3_, a mixture of rotamers) Major rotamer δ 172.4, 171.5, 167.9, 167.9, 155.7, 136.9, 129.4, 128.4, 126.7, 79.9, 59.2, 58.9, 56.2, 52.2, 46.8, 41.1, 37.5, 34.9, 29.7, 28.8, 28.3, 25.0, 24.5, 15.6, 11.6; IR (thin film, neat) *ν*_max_ 3328, 2968, 1748, 1710, 1640, 1497, 1365, 1246, 1174, 1044, 869 cm^−1^; LR-MS (ESI+) *m/z* 561 (M + H^+^); HR-MS (ESI+) calcd for C_29_H_45_N_4_O_7_ (M + H^+^) 561.3283; found 561.3290.

*Allyl (2R,3S)-2-hydroxy-3-methylpentanoate* (**12**). To a solution of d-*allo*-Ile **31** (1.4 g, 10.7 mmol) in dilute sulfuric acid (0.8*N* in H_2_O, 26.7 mL, 21.3 mmol) was added NaNO_2_ (2.2 g, 32.0 mmol) slowly at 0°C. After stirring for 6 h at the same temperature, the reaction mixture was diluted with Et_2_O and extracted with Et_2_O. The combined organic layer was dried over MgSO_4_ and concentrated *in vacuo*. The residue was used in the next step without further purification. To a solution of crude acid **32** (10.7 mmol) and TBAI (0.79 g, 2.13 mmol) in dry DMF (500 mL) were added allyl bromide (1.8 mL, 21.3 mmol) and K_2_CO_3_ (3.0 g, 21.3 mmol) at room temperature. After stirring for overnight, the reaction mixture was quenched with 1*N* HCl and extracted with Et_2_O. The combined organic layer was washed with brine, dried over MgSO_4_, and concentrated *in vacuo*. The residue was purified by flash column chromatography (EtOAc/Hexane = 1:10) to give 1.2 g (65% for 2 steps) of ester **12** as a colorless oil. [α]_D_^20^ = +45.94 (*c* 0.50, CHCl_3_); ^1^H-NMR (800 MHz, CDCl_3_) δ 5.89 (ddt, *J* = 17.1, 10.5, 5.9 Hz, 1H), 5.31 (ddd, *J* = 17.2, 2.9, 1.5 Hz, 1H), 5.24 (dd, *J* = 10.4, 2.3, 1.2 Hz, 1H), 4.67 (ddt, *J* = 13.0, 5.9, 1.2 Hz, 1H), 4.63 (ddt, *J* = 13.1, 5.9, 1.3 Hz, 1H), 4.17 (d, *J* = 3.0 Hz, 1H), 2.61 (s, 1H), 1.83–1.75 (m, 1H), 1.50 (ddq, *J* = 13.8, 7.4, 7.2 Hz, 1H), 1.28 (ddq, *J* = 13.7, 7.5, 7.4 Hz, 1H), 0.91 (t, *J* = 7.5 Hz, 3H), 0.78 (d, *J* = 7.0 Hz, 3H); ^13^C-NMR (200 MHz, CDCl_3_) δ 175.0, 131.5, 119.1, 72.9, 66.0, 38.5, 25.9, 13.1, 11.8; IR (thin film, neat) *ν*_max_ 3524, 2966, 1742, 1461, 1384, 1200, 1138, 934 cm^−1^; LR-MS (ESI+) *m/z* 173 (M + H^+^); HR-MS (ESI+) calcd for C_29_H_45_N_4_O_7_ (M + H^+^) 173.1172; found 173.1163.

*Allyl (R)-2-hydroxy-3-methylbutanoate* (**13**). To a solution of d-valic acid **33** (1.2 g, 10.2 mmol) and TBAI (0.75 g, 2.0 mmol) in dry DMF (25 mL) were added allyl bromide (1.8 mL, 20.3 mmol) and K_2_CO_3_ (2.8 g, 20.3 mmol) at room temperature. After stirring overnight, the reaction mixture was quenched with 1*N* HCl and extracted with Et_2_O. The combined organic layer was washed with brine, dried over MgSO_4_ and concentrated *in vacuo*. The residue was purified by flash column chromatography (EtOAc/Hexane = 1:20) to give 1.5 g (93%) of ester **13** as a colorless oil. [α]_D_^20^ = +24.77 (*c* 1.00, CHCl_3_); ^1^H-NMR (800 MHz, CDCl_3_) δ 5.84 (ddt, *J* = 17.1, 10.6, 5.9 Hz, 1H), 5.26 (dd, *J* = 17.2, 1.3 Hz, 1H), 5.18 (dd, *J* = 10.4, 1.1 Hz, 1H), 4.61 (dd, *J* = 13.1, 5.9 Hz, 1H), 4.57 (dd, *J* = 13.2, 5.9 Hz, 1H), 3.98 (d, *J* = 3.9 Hz, 1H), 2.87 (s, 1H), 2.01 (dtd, *J* = 13.9, 6.9, 3.7 Hz, 1H), 0.94 (d, *J* = 7.3 Hz, 3H), 0.79 (d, *J* = 7.2 Hz, 3H); ^13^C-NMR (200 MHz, CDCl_3_) δ 174.4, 131.4, 118.9, 74.9, 65.8, 32.0, 18.6, 15.8; IR (thin film, neat) *ν*_max_ 3516, 2968, 1743, 1468, 1372, 1197, 1130, 933 cm^−1^; LR-MS (ESI+) *m/z* 181 (M + Na^+^); HR-MS (ESI+) calcd for C_8_H_14_NaO_3_ (M + Na^+^) 181.0835; found 181.0834.

*(S)-2-((tert-Butyldiphenylsilyl)oxy)-3-methylbutanoic acid* (**35**). To a solution of l-valic acid (1.2 g, 10.2 mmol) and DMAP (0.12 g, 1.0 mmol) in dry DMF (5.0 mL) were added imidazole (3.5 g, 50.8 mmol) and TBDPSCl (7.9 mL, 30.5 mmol) at room temperature. After stirring overnight, the reaction mixture was quenched with 1*N* HCl and extracted with Et_2_O. The combined organic layer was washed with brine, dried over MgSO_4_, and concentrated *in vacuo*. The residue was used in the next step without further purification. To a solution of crude silyl ether **34** (10.2 mmol) in a mixture of MeOH and H_2_O (3:1, 40 mL) was added K_2_CO_3_ (2.1 g, 15.2 mmol) at room temperature. After stirring for 1 h, the reaction mixture was quenched with 1*N* HCl and extracted with Et_2_O. The combined organic layer was washed with brine, dried over MgSO_4_ and concentrated *in vacuo*. The residue was purified by flash column chromatography (EtOAc/Hexane = 1:10) to give 1.1 g (92% for 2 steps) of acid **35** as a colorless oil. [α]_D_^20^ = −12.98 (*c* 1.00, CHCl_3_); ^1^H-NMR (800 MHz, CDCl_3_) δ 7.64 (d, *J* = 6.9 Hz, 2H), 7.60 (d, *J* = 6.9 Hz, 2H), 7.42 (dt, *J* = 13.4, 7.4 Hz, 2H), 7.39–7.33 (m, 4H), 4.12 (d, *J* = 3.8 Hz, 1H), 1.95–1.85 (m, 1H), 1.11 (s, 9H), 0.88 (d, *J* = 7.0 Hz, 3H), 0.86 (d, *J* = 6.9 Hz, 3H); ^13^C-NMR (200 MHz, CDCl_3_) δ 174.4, 135.9, 135.8, 132.7, 132.2, 130.2, 130.1, 127.8, 127.8, 77.4, 33.2, 27.0, 19.4, 17.6, 17.3; IR (thin film, neat) *ν*_max_ 2962, 1720, 1471, 1428, 1183, 1113, 1071, 939 cm^−1^; LR-MS (ESI+) *m/z* 355 (M - H^+^); HR-MS (ESI+) calcd for C_21_H_27_OSi (M - H^+^) 355.1735; found 355.1742.

*Allyl (2R,3S)-2-(((S)-2-((tert-Butyldiphenylsilyl)oxy)-3-methylbutanoyl)oxy)-3-methylpentanoate* (**36**). To a solution of acid **35** (591 mg, 1.7 mmol), alcohol **12** (238 mg, 1.4 mmol), and DMAP (338 g, 2.8 mmol) in CH_2_Cl_2_ (14 mL) was added EDC∙HCl (530 g, 2.8 mmol) at room temperature. After stirring for 2 h, the reaction mixture was quenched with 1*N* HCl and extracted with CH_2_Cl_2_. The combined organic layer was washed with aqueous NaHCO_3_, dried over MgSO_4_, and concentrated *in vacuo*. The residue was purified by flash column chromatography (EtOAc/Hexane = 1:20) to give 649 mg (92%) of ester **36** as a colorless oil. [α]_D_^20^ = −23.21 (*c* 1.00, CHCl_3_); ^1^H NMR (800 MHz, CDCl_3_) δ 7.67–7.64 (m, 4H), 7.42–7.38 (m, 2H), 7.36–7.33 (m, 4H), 5.84 (ddt, *J* = 17.2, 10.4, 5.8 Hz, 1H), 5.29 (ddd, *J* = 17.2, 2.9, 1.4 Hz, 1H), 5.21 (ddd, *J* = 10.4, 2.3, 1.1 Hz, 1H), 4.82 (d, *J* = 3.5 Hz, 1H), 4.59 (ddt, *J* = 13.1, 5.7, 1.3 Hz, 1H), 4.52 (ddt, *J* = 13.1, 5.9, 1.3 Hz, 1H), 4.25 (d, *J* = 4.3 Hz, 1H), 2.06–1.96 (m, 1H), 1.90–1.85 (m, 1H), 1.36 (ddq, *J* = 13.8, 7.4, 7.3 Hz, 1H), 1.16 (ddq, *J* = 13.7, 7.6, 7.5 Hz, 1H), 1.14 (d, *J* = 7.5 Hz, 3H), 1.10 (s, 9H), 0.92 (d, *J* = 7.0 Hz, 3H), 0.89 (d, *J* = 6.9 Hz, 3H), 0.86 (t, *J* = 7.5 Hz, 3H); ^13^C-NMR (200 MHz, CDCl_3_) δ 172.0, 169.2, 136.0, 135.9, 133.5, 133.3, 131.7, 129.7, 129.6, 127.5, 127.4, 118.7, 77.6, 75.1, 65.5, 36.7, 33.3, 26.9, 25.7, 19.6, 18.1, 17.1, 14.4, 11.7; IR (thin film, neat) *ν*_max_ 2965, 1756, 1463, 1428, 1192, 1113, 1008, 935, 822 cm^−1^; LR-MS (ESI+) *m/z* 528 (M + NH_4_^+^); HR-MS (ESI+) calcd for C_30_H_46_NO_5_Si (M + NH_4_^+^) 528.3140; found 528.3149.

*Allyl (R)-2-(((S)-2-((tert-Butyldiphenylsilyl)oxy)-3-methylbutanoyl)oxy)-3-methylbutanoate* (**37**). To a solution of acid **35** (741 g, 2.1 mmol), alcohol **13** (274 mg, 1.7 mmol) and DMAP (423 mg, 3.5 mmol) in CH_2_Cl_2_ was added EDC∙HCl (664 mg, 3.5 mmol) at room temperature. After stirring for 2 h, the reaction mixture was quenched with 1*N* HCl, extracted with CH_2_Cl_2_. The combined organic layer was washed with aqueous NaHCO_3_, dried over MgSO_4_ and concentrated *in vacuo*. The residue was purified by flash column chromatography (EtOAc/Hexane = 1:20) to give 809 mg (94%) of ester **37** as a colorless oil. [α]_D_^20^ = +2.49 (*c* 1.00, CHCl_3_); ^1^H-NMR (800 MHz, CDCl_3_) δ 7.64 (td, *J* = 8.4, 1.2 Hz, 4H), 7.40 (tdt, *J* = 7.7, 7.6, 1.3 Hz, 2H), 7.34 (dd, *J* = 7.2, 7.0 Hz, 4H), 5.83 (ddt, *J* = 17.2, 10.4, 5.8 Hz, 1H), 5.28 (dq, *J* = 17.2, 1.4 Hz, 1H), 5.20 (ddt, *J* = 10.4, 1.2, 1.0 Hz, 1H), 4.58 (dt, *J* = 13.1, 5.8, 1.3 Hz, 1H), 4.58 (d, *J* = 4.4 Hz, 1H), 4.51 (ddt, *J* = 13.2, 5.9, 1.2 Hz, 1H), 4.22 (d, *J* = 4.4 Hz, 1H), 2.12–2.06 (m, 1H), 2.05–1.98 (m, 1H), 1.09 (s, 9H), 0.92 (d, *J* = 6.9 Hz, 3H), 0.92 (d, *J* = 6.9 Hz, 3H), 0.90 (d, *J* = 6.9 Hz, 3H), 0.90 (d, *J* = 7.0 Hz, 3H); ^13^C-NMR (200 MHz, CDCl_3_) δ 172.0, 168.9, 136.0, 135.9, 133.4, 133.3, 131.6, 129.7, 129.6, 127.5, 127.4, 118.8, 77.6, 77.0, 65.5, 33.3, 30.1, 26.9, 19.6, 18.5, 18.3, 17.4, 17.2; IR (thin film, neat) *ν*_max_ 2965, 1754, 1471, 1428, 1185, 1113, 990, 934, 822 cm^−1^; LR-MS (ESI+) *m/z* 519 (M + Na^+^); HR-MS (ESI+) calcd for C_29_H_40_NaO_5_Si (M + Na^+^) 519.2537; found 519.2543.

*(2R,3S)-2-(((S)-2-((tert-Butyldiphenylsilyl)oxy)-3-methylbutanoyl)oxy)-3-methylpentanoic acid* (**38**). To a solution of ester **36** (345 mg, 0.7 mmol) and *N*-methylaniline (0.15 mL, 1.4 mmol) in dry THF (6.8 mL) was added Pd(PPh_3_)_4_ (78 mg, 0.1 mmol) at room temperature. After stirring for 2 h, the reaction mixture was quenched with 1*N* HCl and extracted with EtOAc. The combined organic layer was washed with brine, dried over MgSO_4_, and concentrated *in vacuo*. The residue was purified by flash column chromatography through a short pad of silica gel (CH_2_Cl_2_/MeOH = 16:1) to afford 280 mg (88%) of the acid **38** as a yellow oil. [α]_D_^20^ = −15.06 (*c* 1.00, CHCl_3_); ^1^H-NMR (800 MHz, CDCl_3_) δ 7.64–7.62 (m, 4H), 7.42–7.36 (m, 2H), 7.35–7.29 (m, 4H), 4.84 (d, *J* = 3.3 Hz, 1H), 4.25 (d, *J* = 4.1 Hz, 1H), 2.03–1.97 (m, 1H), 1.89 (dtd, *J* = 7.5, 7.0, 3.3 Hz, 1H), 1.36 (ddq, *J* = 13.9, 7.3, 7.1 Hz, 1H), 1.18 (ddq, *J* = 13.8, 7.6, 7.4 Hz, 1H), 1.09 (s, 9H), 0.92 (d, *J* = 7.0 Hz, 3H), 0.91 (d, *J* = 6.9 Hz, 3H), 0.90 (d, *J* = 6.9 Hz, 3H), 0.86 (t, *J* = 7.4 Hz, 3H); ^13^C-NMR (200 MHz, CDCl_3_) δ 174.8, 172.0, 136.0, 135.9, 133.5, 133.3, 129.7, 129.7, 127.5, 127.5, 77.4, 74.4, 36.6, 33.3, 26.9, 25.8, 19.6, 18.4, 17.1, 14.3, 11.7; IR (thin film, neat) *ν*_max_ 2965, 1760, 1730, 1463, 1428, 1182, 1113, 1000, 822 cm^−1^; LR-MS (ESI+) *m/z* 488 (M + NH_4_^+^); HR-MS (ESI+) calcd for C_27_H_42_NO_5_Si (M + NH_4_^+^) 488.2827; found 488.2829.

*(R)-2-(((S)-2-((tert-Butyldiphenylsilyl)oxy)-3-methylbutanoyl)oxy)-3-methylbutanoic acid* (**39**). To a solution of ester **37** (362 mg, 0.7 mmol) and *N*-methylaniline (0.2 mL, 1.5 mmol) in dry THF (7.3 mL) was added Pd(PPh_3_)_4_ (84 mg, 0.1 mmol) at room temperature. After stirring for 2 h, the reaction mixture was quenched with 1*N* HCl and extracted with EtOAc. The combined organic layer was washed with brine, dried over MgSO_4_, and concentrated *in vacuo*. The residue was purified by flash column chromatography through a short pad of silica gel (CH_2_Cl_2_/MeOH = 16:1) to afford 306 mg (92%) of the acid **39** as a yellow oil. [α]_D_^20^ = −11.79 (*c* 1.00, CHCl_3_); ^1^H-NMR (800 MHz, CDCl_3_) δ 7.63 (td, *J* = 8.0, 1.1 Hz, 4H), 7.41–7.36 (m, 2H), 7.32 (dt, *J* = 9.0, 7.6 Hz, 4H), 4.61 (d, *J* = 4.3 Hz, 1H), 4.24 (d, *J* = 4.2 Hz, 1H), 2.14–2.09 (m, 1H), 2.06–1.98 (m, 1H), 1.08 (s, 9H), 0.93 (d, *J* = 7.2 Hz, 3H), 0.92 (d, *J* = 7.0 Hz, 6H), 0.92 (d, *J* = 6.9 Hz, 3H); ^13^C-NMR (200 MHz, CDCl_3_) δ 173.6, 172.0, 136.0, 135.9, 133.4, 133.3, 129.7, 129.7, 127.5, 127.4, 77.4, 76.3, 33.3, 30.0, 26.9, 19.6, 18.6, 18.4, 17.2, 17.1; IR (thin film, neat) *ν*_max_ 2965, 1760, 1726, 1470, 1428, 1181, 1113, 999, 822 cm^−1^; LR-MS (ESI+) *m/z* 474 (M + NH_4_^+^); HR-MS (ESI+) calcd for C_26_H_40_NO_5_Si (M + NH_4_^+^) 474.2670; found 474.2674.

*Izenamide C* (**3**). To a solution of Boc-l**-**Ile*-*Gly*-N*Me-d**-**Phe-l**-**Pro-OMe **5** (34 mg, 0.1 mmol) in CH_2_Cl_2_ (0.8 mL) was added TFA (0.2 mL) dropwise at room temperature. After stirring for 1 h, the reaction mixture was concentrated *in vacuo*. To a solution of above amine salt **40** (0.1 mmol), acid **38** (37 mg, 0.1 mmol), DIPEA (0.1 mL, 0.2 mmol), and HOAt (11 mg, 0.1 mmol) in CH_2_Cl_2_ (1.0 mL) was added EDC∙HCl (24 mg, 0.1 mmol) at room temperature. After stirring overnight, the reaction mixture was quenched with 1*N* HCl and extracted with CH_2_Cl_2_. The combined organic layer was washed with aqueous NaHCO_3_, dried over MgSO_4_, and concentrated *in vacuo*. The residue was used in the next step without further purification. To a solution of crude hexapeptide (0.1 mmol) in THF (1.0 mL) was added TBAF (1M in THF, 0.2 mL, 0.2 mmol) at room temperature. After stirring for 4 h, the reaction mixture was quenched with H_2_O and extracted with EtOAc. The combined organic layer was washed with brine, dried over MgSO_4_, and concentrated *in vacuo*. The residue was purified by flash column chromatography (Acetone/Hexane = 1:3) to give 33 mg (83% for 3 steps) of izenamide C (**3**) as white solid. [α]_D_^20^ = +21.50 (*c* 0.10, MeOH); ^1^H^-^NMR (800 MHz, CD_3_OD, a mixture of rotamers) Major rotamer δ 7.25–7.19 (m, 4H), 7.18–7.14 (m, 1H), 5.57 (t, *J* = 7.6 Hz, 1H), 5.06 (d, *J* = 4.5 Hz, 1H), 4.37 (dd, *J* = 8.3, 5.6 Hz, 1H), 4.33 (d, *J* = 7.0 Hz, 1H), 4.15 (d, *J* = 17.0 Hz, 1H), 4.11 (d, *J* = 4.4 Hz, 1H), 3.90 (d, *J* = 17.0 Hz, 1H), 3.70 (s, 3H), 3.44 (dt, *J* = 10.2, 6.9 Hz, 1H), 3.39 (dt, *J* = 10.2, 5.6 Hz, 1H), 3.19 (dd, *J* = 13.7, 7.8 Hz, 1H), 3.02 (s, 3H), 2.84 (dd, *J* = 13.7, 7.4 Hz, 1H), 2.21–2.17 (m, 1H), 2.11 (qqd, *J* = 6.9, 6.8, 4.5 Hz, 1H), 1.97–1.89 (m, 3H), 1.88–1.81 (m, 2H), 1.50–1.43 (m, 1H), 1.44 (ddq, *J* = 13.8, 7.4, 7.3 Hz, 1H), 1.28 (ddq, *J* = 13.7, 7.6, 7.5 Hz, 1H), 1.18–1.11 (m, 1H), 1.01 (d, *J* = 6.9 Hz, 3H), 0.95 (t, *J* = 6.7 Hz, 3H), 0.95 (d, *J* = 6.5 Hz, 3H), 0.93 (d, *J* = 6.9 Hz, 3H), 0.91 (d, *J* = 6.8 Hz, 3H), 0.89 (t, *J* = 7.4 Hz, 3H); ^13^C-NMR (200 MHz, CD_3_OD, a mixture of rotamers) Major rotamer δ 175.2, 174.0, 173.4, 172.2, 170.2, 170.1, 138.6, 130.5, 129.4, 127.6, 78.0, 76.5, 60.6, 58.9, 57.8, 52.7, 48.3, 41.9, 38.5, 37.9, 35.7, 33.3, 30.5, 29.9, 27.0, 26.0, 25.7, 19.4, 16.9, 16.1, 14.7, 11.8, 11.4; IR (thin film, neat) *ν*_max_ 3317, 2960, 2877, 1744, 1637, 1519, 1447, 1199, 1176, 1137, 1030, 753 cm^−1^; LR-MS (ESI+) *m/z* 675 (M + H^+^); HR-MS (ESI+) calcd for C_35_H_55_N_4_O_9_ (M + H^+^) 675.3964; found 675.3949.

*Pentapeptide* (**42**). To a solution of Boc-l**-**Ile*-*l-Ala*-N*Me-d-Phe-l**-**Pro-OMe **4** (140 mg, 0.2 mmol) in CH_2_Cl_2_ (1.6 mL) was added TFA (0.4 mL) dropwise at room temperature. After stirring for 1 h, the reaction mixture was concentrated *in vacuo*. The residue was used in the next step without further purification. To a solution of above amine salt **41** (0.2 mmol), acid **6** (100 mg, 0.3 mmol), DIPEA (0.1 mL, 0.7 mmol), and HOAt (43 mg, 0.3 mmol) in CH_2_Cl_2_ (2.4 mL) was added EDC∙HCl (93 mg, 0.5 mmol) at room temperature. After stirring overnight, the reaction mixture was quenched with 1*N* HCl and extracted with CH_2_Cl_2_. The combined organic layer was washed with aqueous NaHCO_3_, dried over MgSO_4_, and concentrated *in vacuo*. The residue was purified by flash column chromatography (Acetone/Hexane = 1:3) to give 145 mg (77% for 2 steps) of pentapeptide **42** as white solid. [α]_D_^20^ = +67.88 (*c* 0.50, CHCl_3_); ^1^H-NMR (800 MHz, CDCl_3_, a mixture of rotamers) Major rotamer δ 7.23–7.18 (m, 4H), 7.16 (t, *J* = 7.2 Hz, 1H), 6.64 (s, 1H), 6.51 (d, *J* = 6.8 Hz, 1H), 5.66 (dd, *J* = 9.5, 6.5 Hz, 1H), 4.72 (dq, *J* = 7.2, 7.0 Hz, 1H), 4.44 (dd, *J* = 8.1, 6.7 Hz, 1H), 4.27–4.24 (m, 1H), 4.22–4.19 (m, 1H), 3.71 (s, 3H), 3.65–3.60 (m, 1H), 3.48–3.42 (m, 1H), 3.21 (dt, *J* = 10.6, 7.4 Hz, 1H), 3.19 (dd, *J* = 14.5, 6.4 Hz, 1H), 2.98 (s, 3H), 2.95 (dd, *J* = 14.4, 9.6 Hz, 1H), 2.58–2.51 (m, 1H), 2.48–2.40 (m, 1H), 2.23–2.18 (m, 1H), 1.93–1.87 (m, 1H), 1.87–1.75 (m, 4H), 1.61 (s, 3H), 1.52–1.46 (m, 5H), 1.46 (s, 9H), 1.46–1.42 (m, 1H), 1.12–1.02 (m, 1H), 0.90 (d, *J* = 6.2 Hz, 3H), 0.88 (d, *J* = 6.8 Hz, 3H), 0.85 (t, *J* = 7.4 Hz, 3H), 0.83 (d, *J* = 6.8 Hz, 3H), 0.82 (d, *J* = 6.9 Hz, 3H); ^13^C-NMR (200 MHz, CDCl_3_) δ 172.6, 172.4, 172.2, 169.8, 168.1, 156.3, 136.6, 129.5, 128.3, 126.7, 93.8, 79.3, 70.4, 59.3, 57.7, 55.6, 52.3, 52.2, 46.9, 45.2, 41.6, 40.1, 37.5, 34.7, 30.9, 30.3, 28.8, 28.4, 25.3, 24.9, 24.8, 24.8, 23.1, 22.1, 17.6, 15.3, 11.4; IR (thin film, neat) *ν*_max_ 2961, 2875, 1751, 1700, 1632, 1532, 1455, 1366, 1257, 1175, 1047, 859 cm^−1^; LR-MS (ESI+) *m/z* 789 (M + NH_4_^+^); HR-MS (ESI+) calcd for C_41_H_69_N_6_O_9_ (M + NH_4_^+^) 789.5121; found 789.5134.

*Izenamide A* (**1**). To a solution of pentapeptide **42** (41 mg, 0.1 mmol) in CH_2_Cl_2_ (0.8 mL) was added TFA (0.2 mL) dropwise at room temperature. After stirring for 1 h, the reaction mixture was concentrated *in vacuo*. To a solution of above amine salt **43** (0.1 mmol), acid **39** (32 mg, 0.1 mmol), DIPEA (0.1 mL, 0.2 mmol), and HOAt (10 mg, 0.1 mmol) in CH_2_Cl_2_ (1.0 mL) was added EDC∙HCl (21 mg, 0.1 mmol) at room temperature. After stirring for 6 h, the reaction mixture was quenched with 1*N* HCl and extracted with CH_2_Cl_2_. The combined organic layer was washed with aqueous NaHCO_3_, dried over MgSO_4_ and concentrated *in vacuo*. The residue was used in the next step without further purification. To a solution of crude heptapeptide (0.1 mmol) in THF (1.0 mL) was added TBAF (1M in THF, 0.2 mL, 0.2 mmol) at room temperature. After stirring for 6 h, the reaction mixture was quenched with H_2_O and extracted with EtOAc. The combined organic layer was washed with brine, dried over MgSO_4_ and concentrated *in vacuo*. The residue was purified by flash column chromatography (Acetone/Hexane = 1:3 to 1:1) to give 29 mg (66% for 3 steps) of izenamide A (**1**) as white solid. [α]_D_^20^ = −17.15 (*c* 0.20, MeOH); ^1^H-NMR (800 MHz, CD_3_OD, a mixture of rotamers) Major rotamer δ 7.26–7.19 (m, 4H), 7.18–7.13 (m, 1H), 5.69 (dd, *J* = 10.0, 5.9 Hz, 1H), 4.71 (d, *J* = 5.9 Hz, 1H), 4.68 (q, *J* = 7.1 Hz, 1H), 4.41 (dd, *J* = 7.7, 7.4 Hz, 1H), 4.19 (d, *J* = 7.6 Hz, 1H), 4.11 (d, *J* = 4.4 Hz, 1H), 4.00–3.95 (m, 2H), 3.71 (s, 3H), 3.47–3.45 (m, 1H), 3.42–3.37 (m, 1H), 3.11 (dd, *J* = 14.3, 5.9 Hz, 1H), 3.06 (s, 3H), 2.93 (dd, *J* = 14.3, 10.0 Hz, 1H), 2.41–2.34 (m, 2H), 2.29–2.23 (m, 1H), 2.17–2.12 (m, 1H), 2.11–2.07 (m, 1H), 1.97–1.90 (m, 1H), 1.87–1.82 (m, 2H), 1.80–1.73 (m, 1H), 1.60–1.52 (m, 2H), 1.48 (ddd, *J* = 13.4, 7.6, 3.4 Hz, 1H), 1.31 (ddd, *J* = 9.3, 7.5, 4.3 Hz, 1H), 1.13 (ddd, *J* = 13.6, 9.5, 7.3 Hz, 1H), 1.01 (d, *J* = 6.8 Hz, 3H), 1.00 (d, *J* = 6.8 Hz, 6H), 0.92 (d, *J* = 6.8 Hz, 3H), 0.92 (d, *J* = 6.5 Hz, 3H), 0.89 (d, *J* = 6.3 Hz, 3H), 0.87 (t, *J* = 7.4 Hz, 3H), 0.86 (d, *J* = 6.7 Hz, 3H), 0.82 (d, *J* = 7.0 Hz, 3H); ^13^C-NMR (200 MHz, CD_3_OD, a mixture of rotamers) Major rotamer δ 175.8, 174.6, 173.9, 173.8, 173.0, 171.8, 170.4, 138.3, 130.8, 129.3, 127.6, 80.6, 76.4, 71.3, 60.9, 58.7, 57.2, 52.6, 52.2, 48.5, 46.6, 41.4, 41.4, 38.3, 35.6, 33.3, 31.7, 31.1, 30.0, 26.2, 26.0, 25.7, 23.8, 22.2, 19.3, 19.2, 18.1, 17.2, 16.6, 15.8, 11.4; IR (thin film, neat) *ν*_max_ 3318, 2960, 2878, 1742, 1637, 1544, 1450, 1366, 1265, 1198, 1038, 753 cm^−1^; LR-MS (ESI+) *m/z* 854 (M + Na^+^); HR-MS (ESI+) calcd for C_43_H_69_N_5_NaO_11_ (M + Na^+^) 854.4886; found 854.4888.

*Izenamide B* (**2**). To a solution of pentapeptide **42** (46 mg, 0.1 mmol) in CH_2_Cl_2_ (0.8 mL) was added TFA (0.2 mL) dropwise at room temperature. After stirring for 1 h, the reaction mixture was concentrated *in vacuo*. To a solution of above amine salt **43** (0.1 mmol), acid **38** (36 mg, 0.1 mmol), DIPEA (0.1 mL, 0.2 mmol), and HOAt (11 mg, 0.1 mmol) in CH_2_Cl_2_ (1.0 mL) was added EDC∙HCl (23 mg, 0.1 mmol) at room temperature. After stirring for 6 h, the reaction mixture was quenched with 1*N* HCl and extracted with CH_2_Cl_2_. The combined organic layer was washed with aqueous NaHCO_3_, dried over MgSO_4_, and concentrated *in vacuo*. The residue was used in the next step without further purification. To a solution of crude heptapeptide (0.1 mmol) in THF (1.0 mL) was added TBAF (1M in THF, 0.2 mL, 0.2 mmol) at room temperature. After stirring for 6 h, the reaction mixture was quenched with H_2_O and extracted with EtOAc. The combined organic layer was washed with brine, dried over MgSO_4_, and concentrated *in vacuo*. The residue was purified by flash column chromatography (Acetone/Hexane = 1:3 to 1:1) to give 31 mg (61% from **S5**) of izenamide B (**2**) as white solid. [α]_D_^20^ = −11.62 (*c* 1.30, MeOH); ^1^H-NMR (800 MHz, CD_3_OD) δ 7.25–7.19 (m, 4H), 7.18–7.13 (m, 1H), 5.69 (dd, *J* = 10.0, 5.9 Hz, 1H), 4.93 (d, *J* = 4.4 Hz, 1H), 4.67 (q, *J* = 7.0 Hz, 1H), 4.41 (dd, *J* = 7.8, 7.3 Hz, 1H), 4.19 (d, *J* = 7.5 Hz, 1H), 4.13 (d, *J* = 4.3 Hz, 1H), 4.00–3.96 (m, 2H), 3.71 (s, 3H), 3.48–3.44 (m, 1H), 3.42–3.38 (m, 1H), 3.11 (dd, *J* = 14.3, 5.9 Hz, 1H), 3.06 (s, 3H), 2.93 (dd, *J* = 14.3, 10.0 Hz, 1H), 2.41–2.33 (m, 2H), 2.28–2.23 (m, 1H), 2.11–2.07 (m, 1H), 1.96–1.89 (m, 2H), 1.88–1.81 (m, 2H), 1.79–1.74 (m, 1H), 1.59–1.53 (m, 2H), 1.51–1.43 (m, 2H), 1.33–1.27 (m, 2H), 1.18–1.10 (m, 1H), 1.01 (d, *J* = 6.9 Hz, 3H), 0.97 (d, *J* = 6.9 Hz, 3H), 0.95 (t, *J* = 7.5 Hz, 3H), 0.92 (d, *J* = 6.4 Hz, 3H), 0.92 (d, *J* = 6.8 Hz, 3H), 0.89 (d, *J* = 6.2 Hz, 3H), 0.87 (t, *J* = 7.4 Hz, 3H), 0.86 (d, *J* = 6.8 Hz, 3H), 0.82 (d, *J* = 7.1 Hz, 3H); ^13^C-NMR (200 MHz, CD_3_OD, a mixture of rotamers) Major rotamer δ 175.7, 174.6, 173.9, 173.8, 173.0, 171.9, 170.3, 138.3, 130.8 129.3 127.6, 78.3, 76.4, 71.4, 60.9, 58.7, 57.2, 52.6, 52.3, 48.5, 46.6, 41.5, 41.4, 38.4, 38.3, 35.6, 33.3, 31.0, 30.0, 27.2, 26.2, 26.1, 25.7, 23.8, 22.2, 19.3, 17.1, 16.6, 15.8, 14.7, 11.9, 11.5; IR (thin film, neat) *ν*_max_ 3308, 2960, 2877, 1743, 1636, 1533, 1448, 1361, 1265, 1195, 1038, 753 cm^−1^; LR-MS (ESI+) *m/z* 846 (M + H^+^); HR-MS (ESI+) calcd for C_44_H_72_N_5_O_11_ (M + H^+^) 846.5223; found 846.5229.

## 4. Conclusions

In conclusion, we have accomplished the first total syntheses of izenamides A (**1**), B (**2**), and C (**3**) and confirmed the stereochemistry of izenamide B, which was originally suggested by Suenaga and coworkers. The key features of the syntheses include the DKP formation-free amide coupling sequence and amidation with minimal epimerization. Our versatile and convergent synthesis of izenamides is expected to be widely utilized for the efficient synthesis of izenamides and their analogs for the development of novel cathepsin D inhibitors. Further studies on the structure-activity relationship of izenamides are currently underway and successful results will be reported in due course.

## Supplementary Materials

The following are available online. The ^1^H and ^13^C NMR spectra of **16**, **19**, **20**, **24**, **25**, **4**, **5**, **12**, **13**, **35**, **36**, **37**, **38**, **39**, **3**, **42**, **1** and **2**.

## References

[1] Benes P., Vetvicka V., Fusek M. (2008). Cathepsin D—many functions of one aspartic protease. Crit. Rev. Oncol. Hematol..

[2] Vidoni C., Follo C., Savino M., Melone M.A., Isidoro C. (2016). The role of cathepsin d in the pathogenesis of human neurodegenerative disorders. Med. Res. Rev..

[3] Loscalzo J. (1996). The Macrophage and Fibrinolysis.

[4] Simon D.I., Ezratty A.M., Loscalzo J. (1994). The fibrin (ogen) olytic properties of cathepsin d. Biochemistry.

[5] Koike M., Shibata M., Ohsawa Y., Nakanishi H., Koga T., Kametaka S., Waguri S., Momoi T., Kominami E., Peters C. (2003). Involvement of two different cell death pathways in retinal atrophy of cathepsin D-deficient mice. Mol. Cell. Neurosci..

[6] Saftig P., Hetman M., Schmahl W., Weber K., Heine L., Mossmann H., Köster A., Hess B., Evers M., von Figura K. (1995). Mice deficient for the lysosomal proteinase cathepsin D exhibit progressive atrophy of the intestinal mucosa and profound destruction of lymphoid cells. EMBO J..

[7] Baechle D., Flad T., Cansier A., Steffen H., Schittek B., Tolson J., Herrmann T., Dihazi H., Beck A., Mueller G.A. (2006). Cathepsin D is present in human eccrine sweat and involved in the postsecretory processing of the antimicrobial peptide DCD-1L. J. Biol. Chem..

[8] Hakala J.K., Oksjoki R., Laine P., Du H., Grabowski G.A., Kovanen P.T., Pentikäinen M.O. (2003). Lysosomal enzymes are released from cultured human macrophages, hydrolyze LDL in vitro, and are present extracellularly in human atherosclerotic lesions. Arterioscler. Thromb. Vasc. Biol..

[9] Bańkowska A., Gacko M., Chyczewska E., Worowska A. (1997). Biological and diagnostic role of cathepsin D. Rocz. Akad. Med. Bialymst..

[10] Vetvicka V., Vetvickova J., Fusek M. (1999). Anti-human procathepsin D activation peptide antibodies inhibit breast cancer development. Breast Cancer Res. Treat..

[11] Vetvicka V., Vetvickova J., Hilgert I., Voburka Z., Fusek M. (1997). Analysis of the interaction of procathepsin D activation peptide with breast cancer cells. Int. J. Cancer.

[12] Vignon F., Capony F., Ghambon M., Freiss G., Garcia M., Rochefort H. (1986). Autocrine growth stimulation of the MCF 7 breast cancer cells by the estrogen-regulated 52 K protein. Endocrinology.

[13] Haidar B., Kiss R.S., Sarov-Blat L., Brunet R., Harder C., McPherson R., Marcel Y.L. (2006). Cathepsin D, a lysosomal protease, regulates ABCA1-mediated lipid efflux. J. Biol. Chem..

[14] Elliott D.A., Tsoi K., Holinkova S., Chan S.L., Kim W.S., Halliday G.M., Rye K.-A., Garner B. (2011). Isoform-specific proteolysis of apolipoprotein-E in the brain. Neurobiol. Aging.

[15] Mutka A.L., Haapanen A., Käkelä R., Lindfors M., Wright A.K., Inkinen T., Hermansson M., Rokka A., Corthals G., Jauhiainen M. (2010). Murine cathepsin D deficiency is associated with dysmyelination/myelin disruption and accumulation of cholesteryl esters in the brain. J. Neurochem..

[16] Zhou W., Scott S., Shelton S., Crutcher K. (2006). Cathepsin D-mediated proteolysis of apolipoprotein E: Possible role in Alzheimer’s disease. Neuroscience.

[17] Myllykangas L., Tyynelä J., Page-McCaw A., Rubin G.M., Haltia M.J., Feany M.B. (2005). Cathepsin D-deficient drosophila recapitulate the key features of neuronal ceroid lipofuscinoses. Neurobiol. Dis..

[18] Koike M., Nakanishi H., Saftig P., Ezaki J., Isahara K., Ohsawa Y., Schulz-Schaeffer W., Watanabe T., Waguri S., Kametaka S. (2000). Cathepsin D deficiency induces lysosomal storage with ceroid lipofuscin in mouse CNS neurons. J. Neurosci..

[19] Ramirez-Montealegre D., Rothberg P.G., Pearce D.A. (2006). Another disorder finds its gene. Brain.

[20] Umezawa H., Aoyagi T., Morishima H., Matsuzaki M., Hamada M., Takeuchi T. (1970). Pepstatin, a new pepsin inhibitor produced by agtinomygetes. J. Antibiot..

[21] Kwan J.C., Eksioglu E.A., Liu C., Paul V.J., Luesch H. (2009). Grassystatins A−C from marine cyanobacteria, potent cathepsin E inhibitors that reduce antigen presentation. J. Med. Chem..

[22] Al-Awadhi F.H., Law B.K., Paul V.J., Luesch H. (2017). Grassystatins D–F, potent aspartic protease inhibitors from marine cyanobacteria as potential antimetastatic agents targeting invasive breast cancer. J. Nat. Prod..

[23] Williams P.G., Yoshida W.Y., Moore R.E., Paul V.J. (2003). The isolation and structure elucidation of tasiamide B, a 4-amino-3-hydroxy-5-phenylpentanoic acid containing peptide from the marine cyanobacterium symploca sp.. J. Nat. Prod..

[24] Al-Awadhi F.H., Ratnayake R., Paul V.J., Luesch H. (2016). Tasiamide F, a potent inhibitor of cathepsins D and E from a marine cyanobacterium. Bioorgan. Med. Chem..

[25] Kanamori Y., Iwasaki A., Sumimoto S., Matsubara T., Sato T., Suenaga K. (2018). Izenamides A and B, statine-containing depsipeptides, and an analogue from a marine cyanobacterium. J. Nat. Prod..

[26] Ghosh A.K., Bischoff A., Cappiello J. (2003). Asymmetric total synthesis of the gastroprotective microbial agent AI-77-B. Eur. J. Org. Chem..

[27] Veeresha G., Datta A. (1997). Stereoselective synthesis of (−)-*N*-Boc-statine and (−)-*N*-Boc-norstatine. Tetrahedron Lett..

[28] Fernández-Llamazares A.I., Spengler J., Albericio F. (2015). Review backbone *N*-modified peptides: How to meet the challenge of secondary amine acylation. Biopolymers.

[29] Dai L., Chen B., Lei H., Wang Z., Liu Y., Xu Z., Ye T. (2012). Total synthesis and stereochemical revision of lagunamide A. Chem. Commun..

