# Supplementary material for: Total Syntheses of Cathepsin D Inhibitory Izenamides A, B, and C and Structural Confirmation of Izenamide B
Source: Molecules. 2019 Sep 20;24(19):3424. doi: 10.3390/molecules24193424 (PMC6804045; doi:10.3390/molecules24193424)

Supporting Information

Total Syntheses of Cathepsin D Inhibitory Izenamides A, B, and C and Structural Confirmation of Izenamide B

Changjin Lim [1,*]

[1] College of Pharmacy, CHA University, 120 Haeryong-ro, Pocheon 11160, Gyeonggi-do, Republic of Korea
* Correspondence: koryoi@cha.ac.kr; Tel.: +82-31-881-7193 (C.L.)

Table of Contents

I. ^{1}H- and ^{13}C-NMR Spectra ··· S2

^{1}H- and ^{13}C-NMR Spectra of **16**

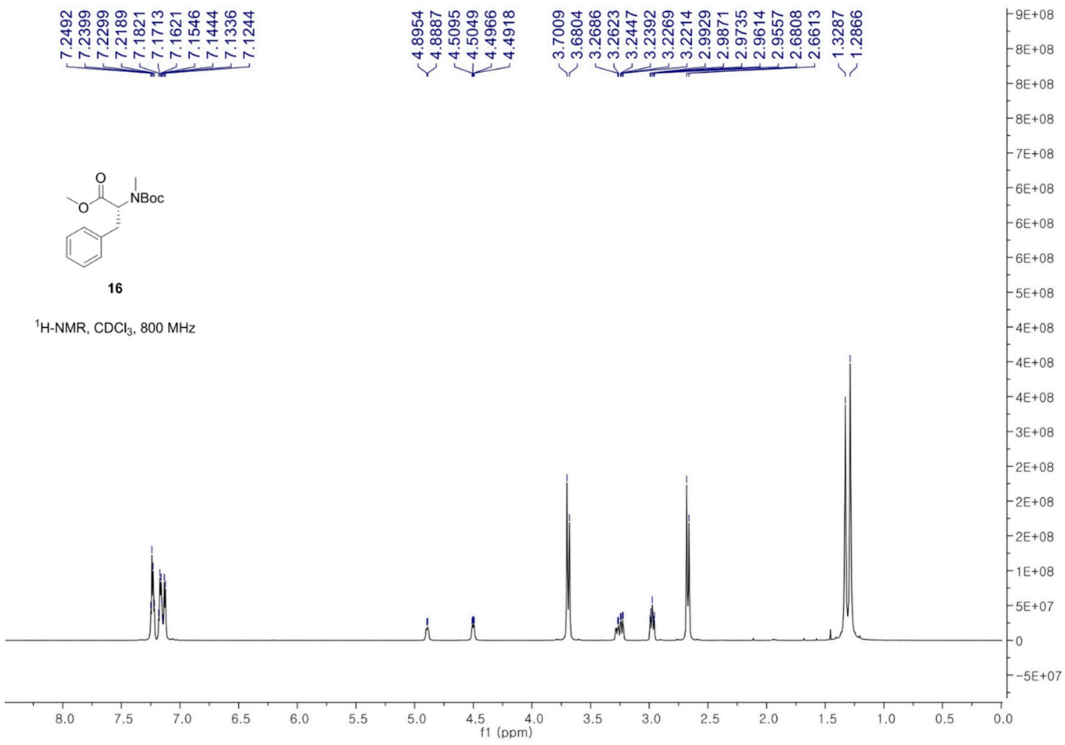

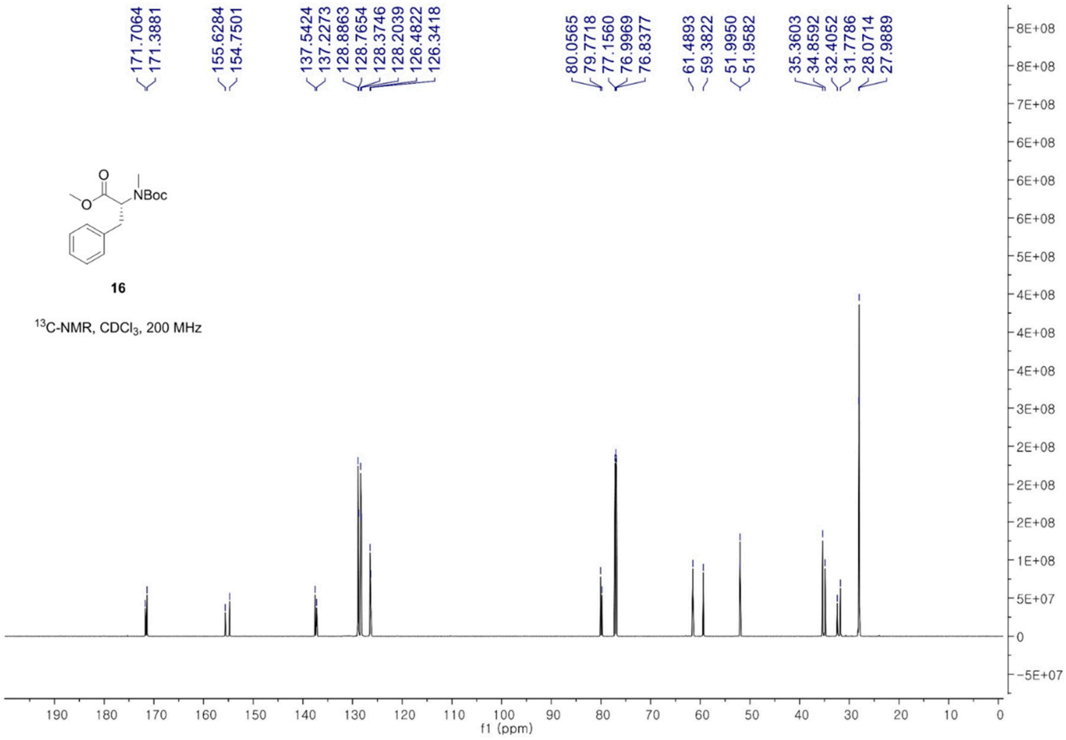

^{1}H- and ^{13}C-NMR Spectra of **19**

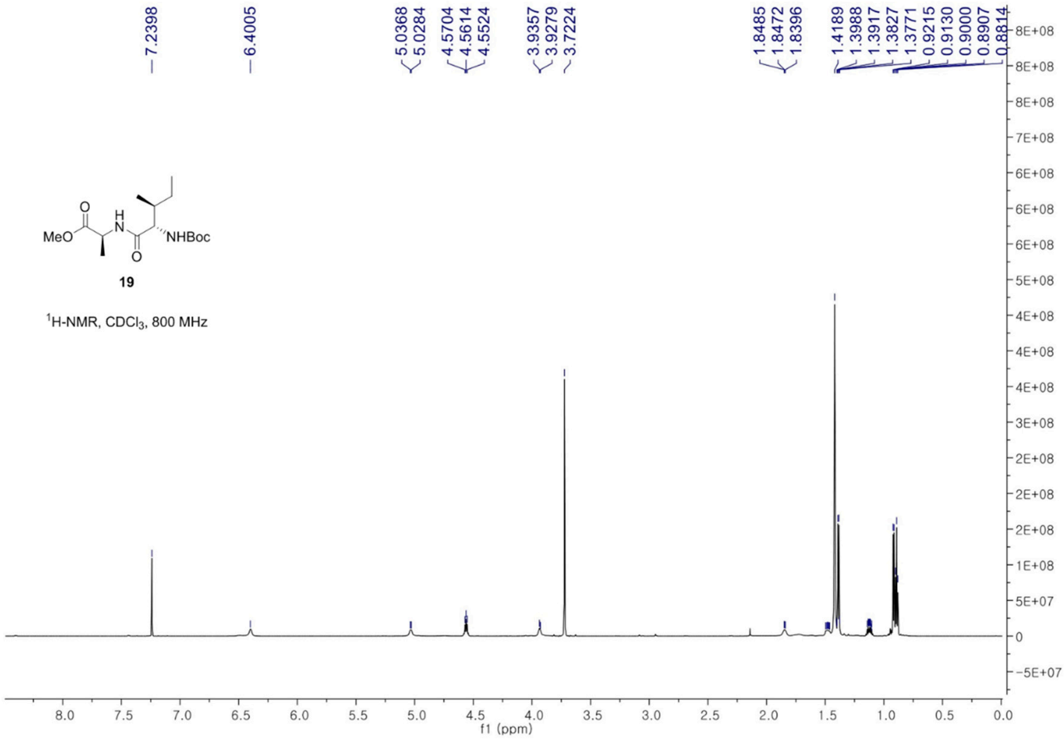

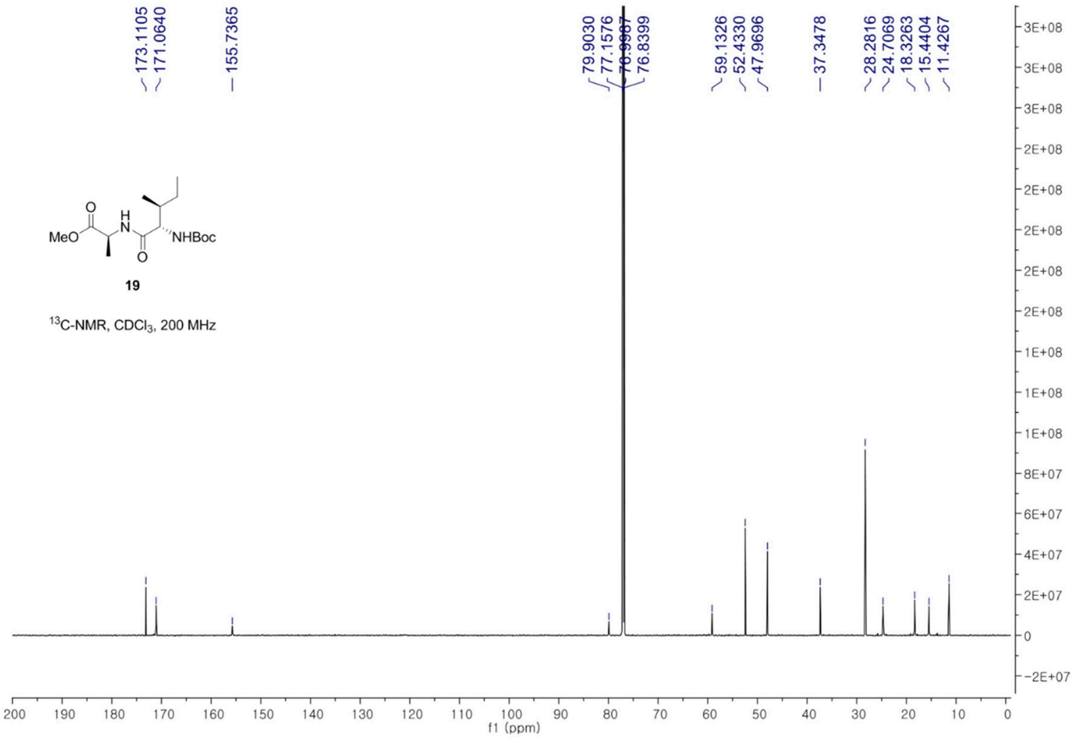

^{1}H- and ^{13}C-NMR Spectra of **20**

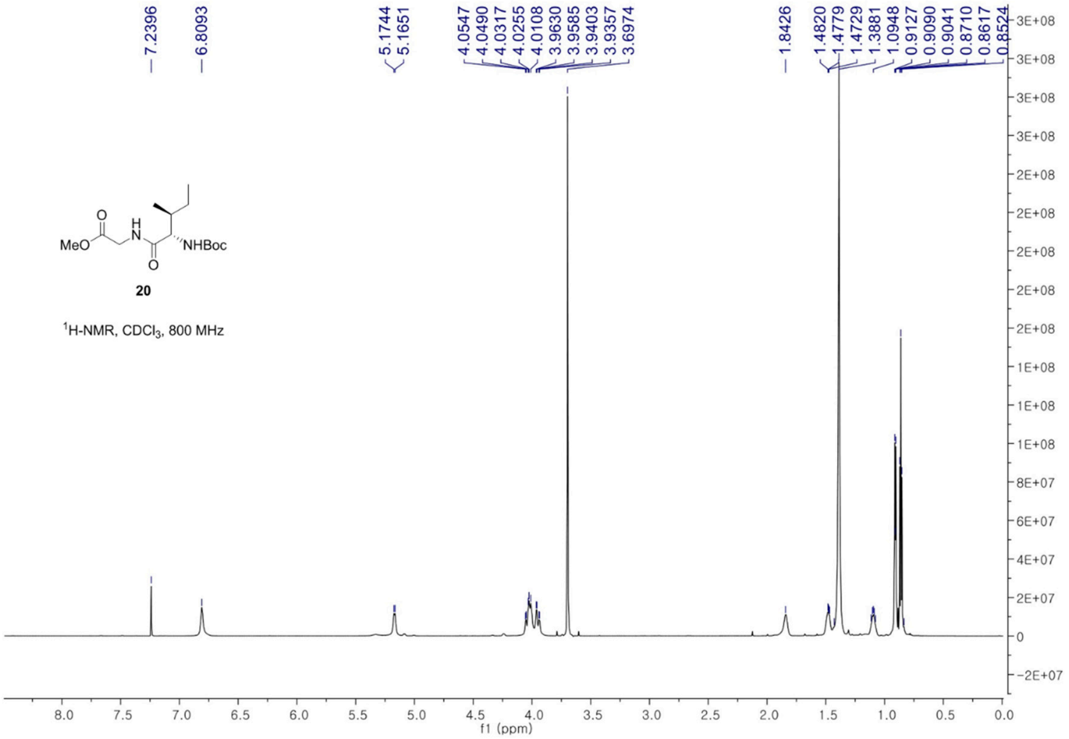

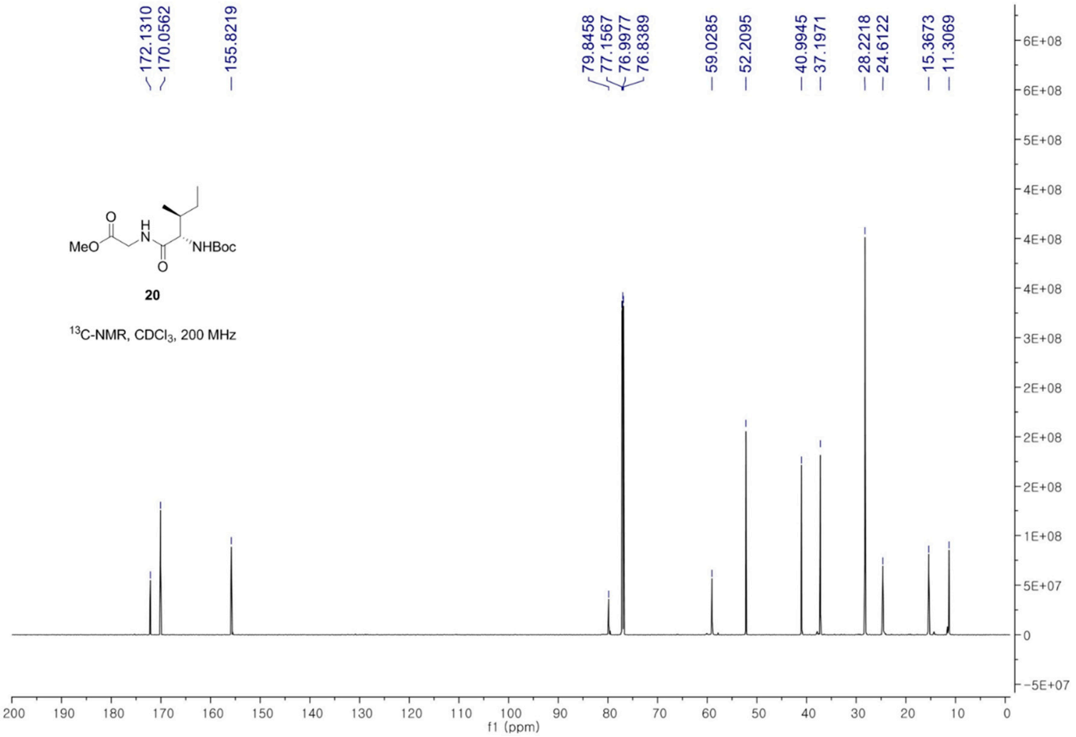

w

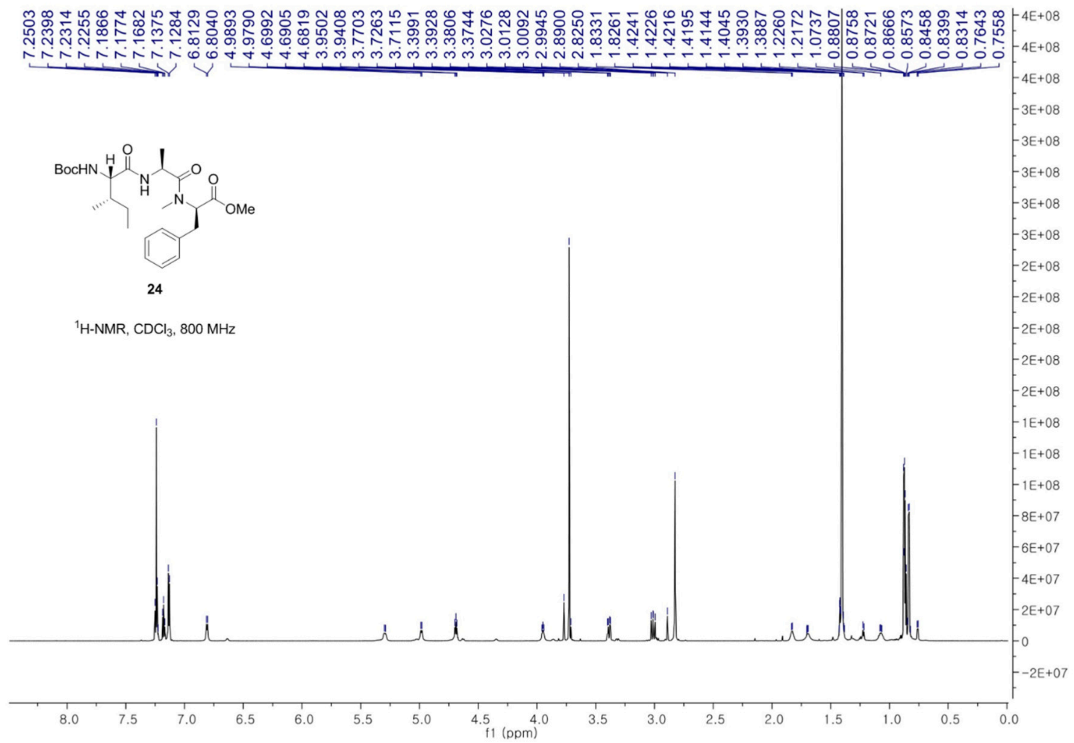

24

^{1}H-NMR, $CDCl_3$, 800 MHz

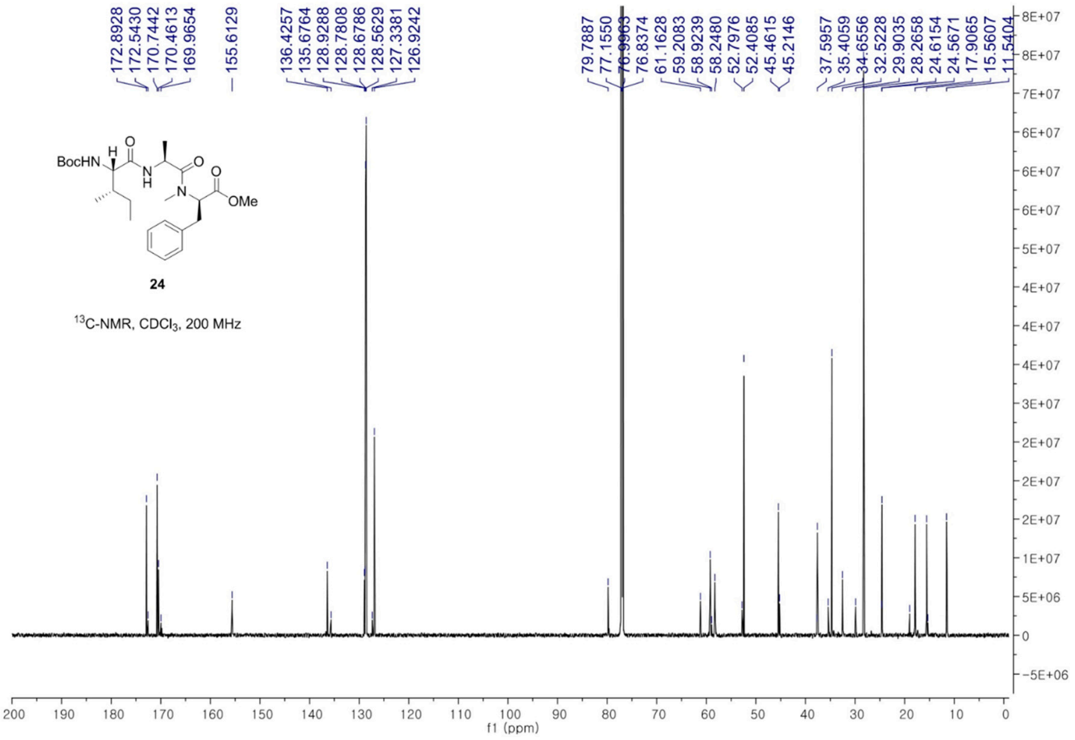

24

^{13}C-NMR, $CDCl_3$, 200 MHz

^{1}H- and ^{13}C-NMR Spectra of **25**

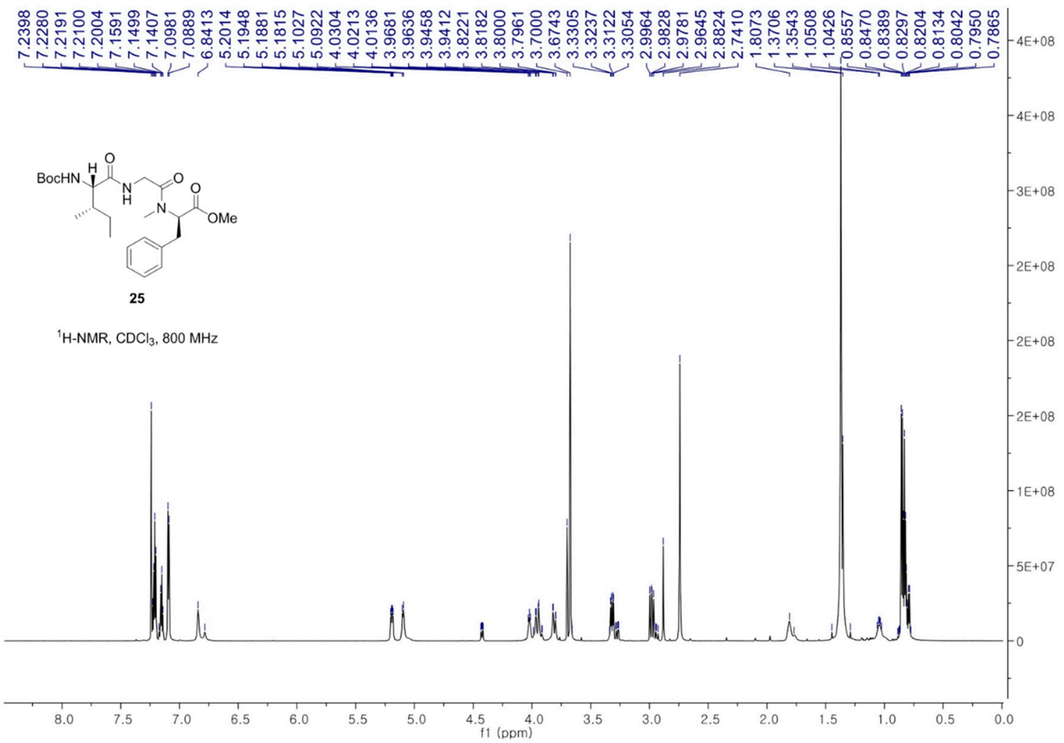

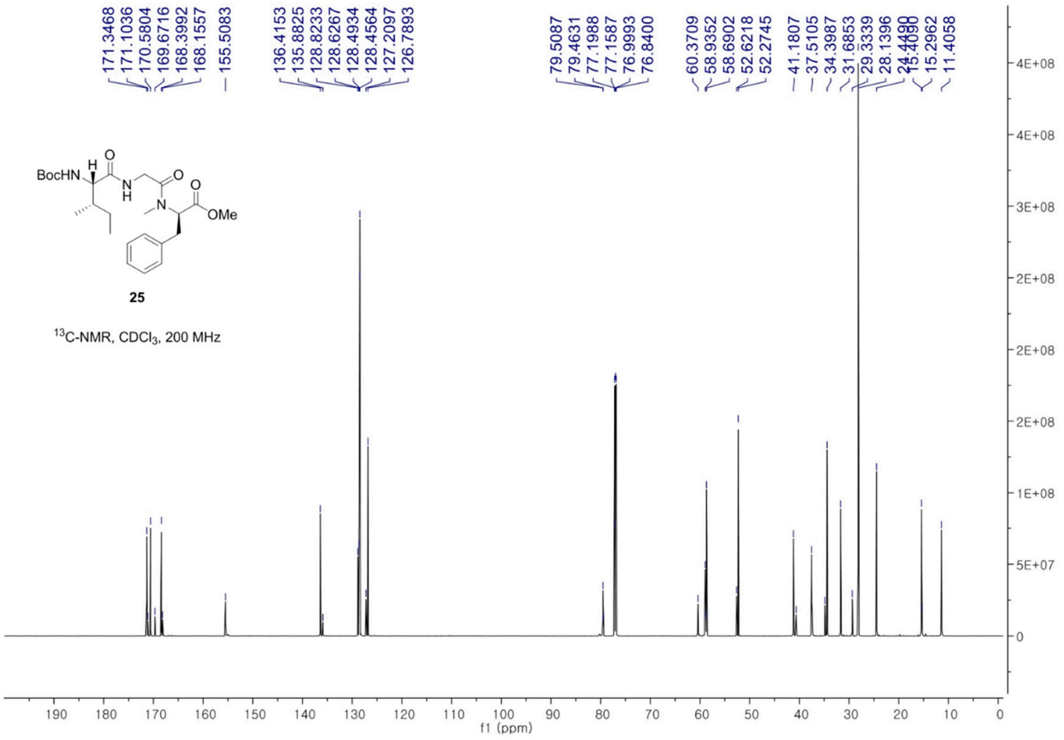

^{1}H- and ^{13}C-NMR Spectra of **4**

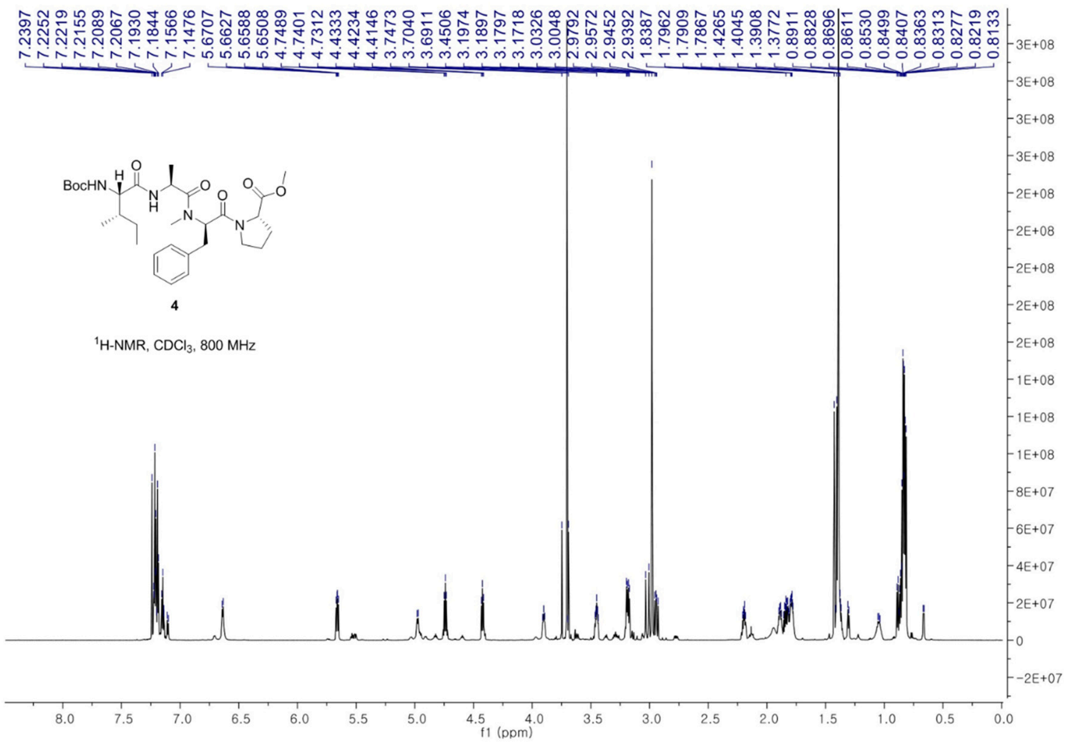

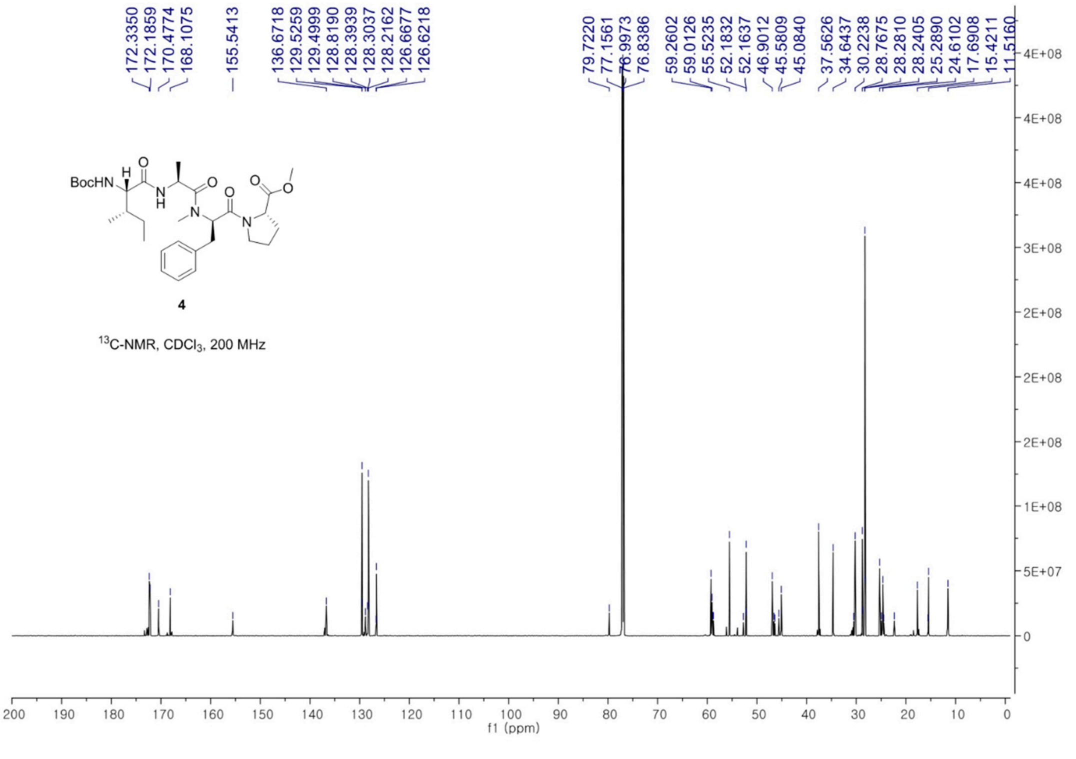

^{1}H- and ^{13}C-NMR Spectra of **5**

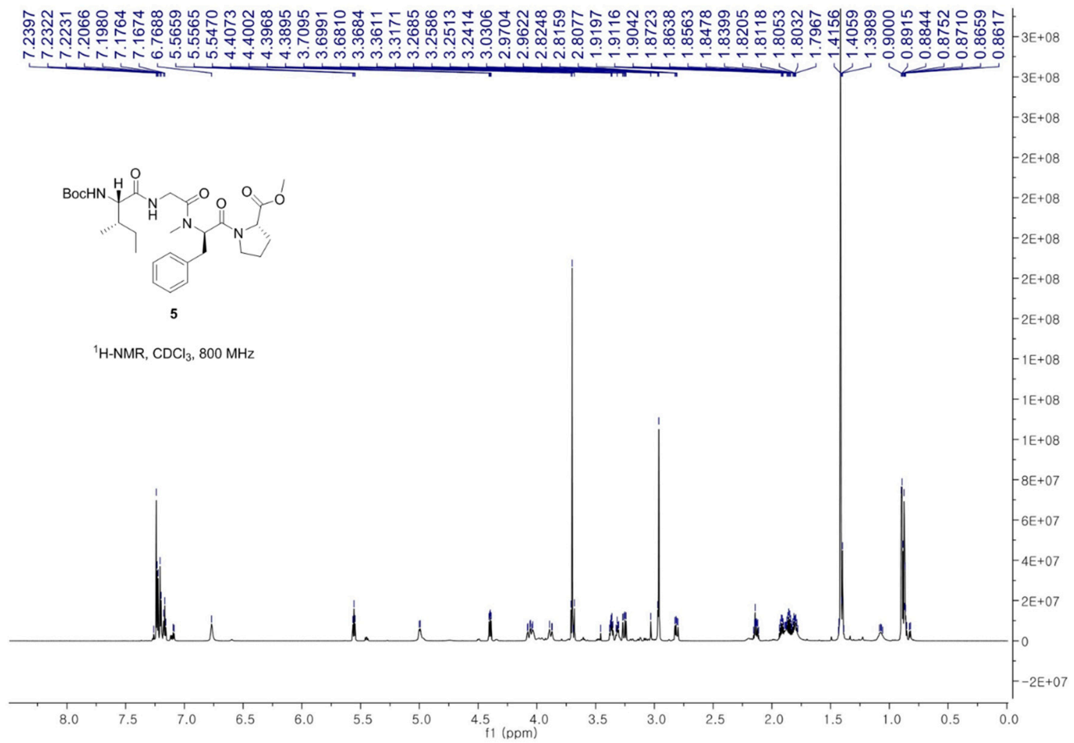

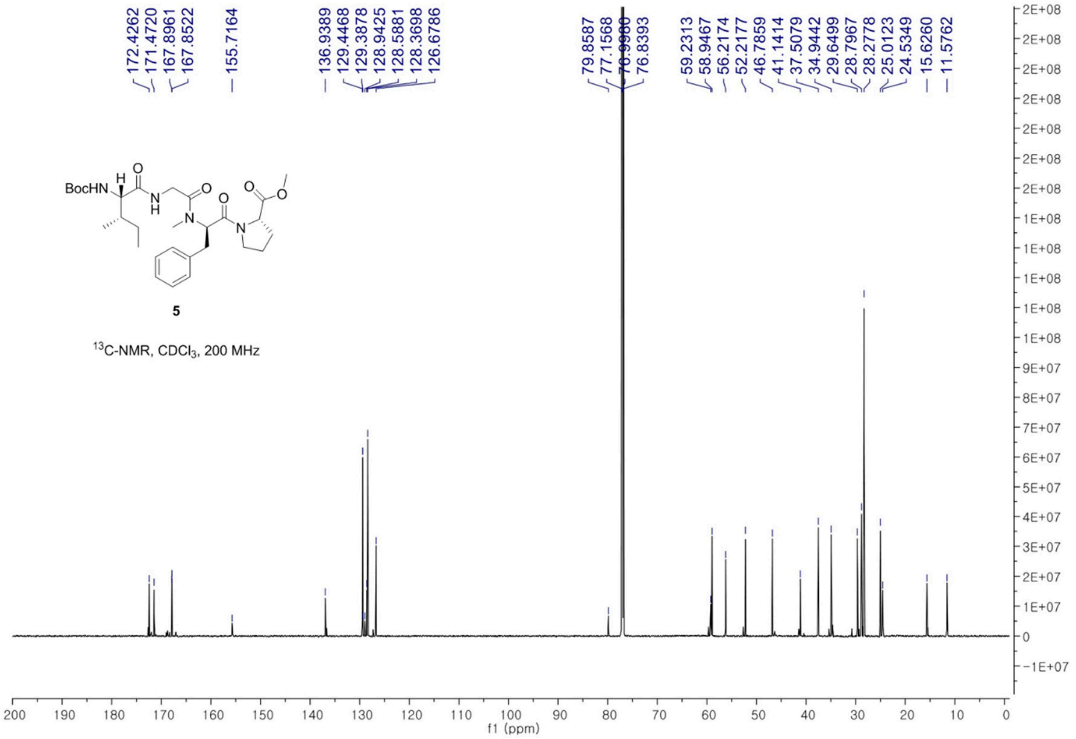

^{1}H- and ^{13}C-NMR Spectra of **12**

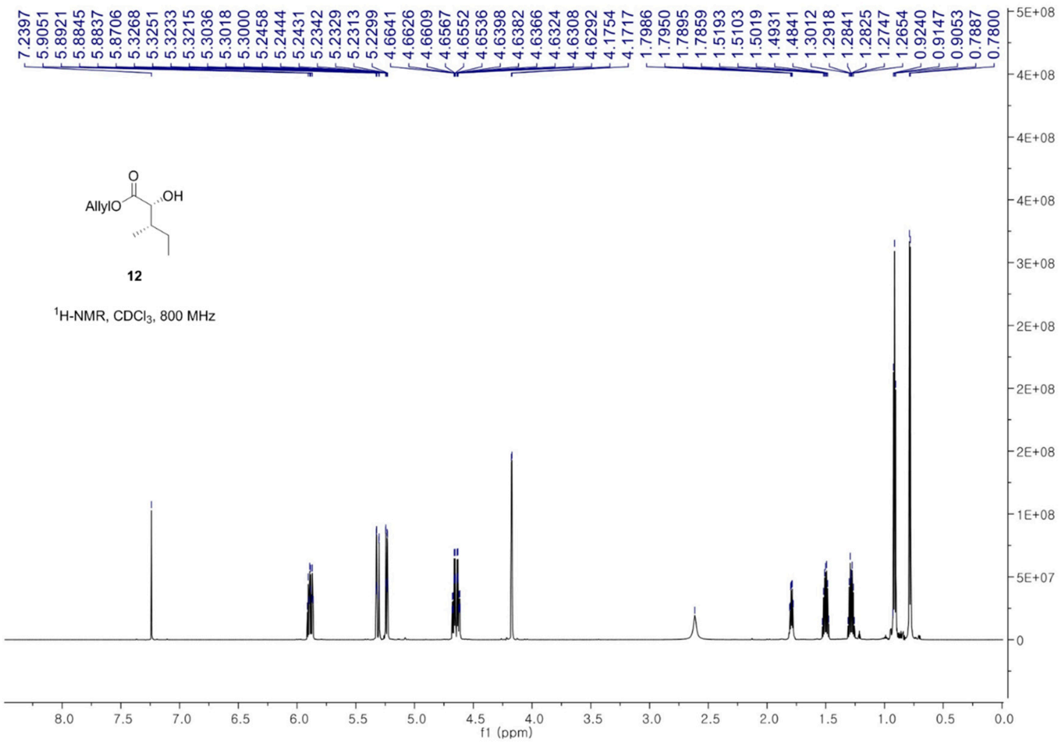

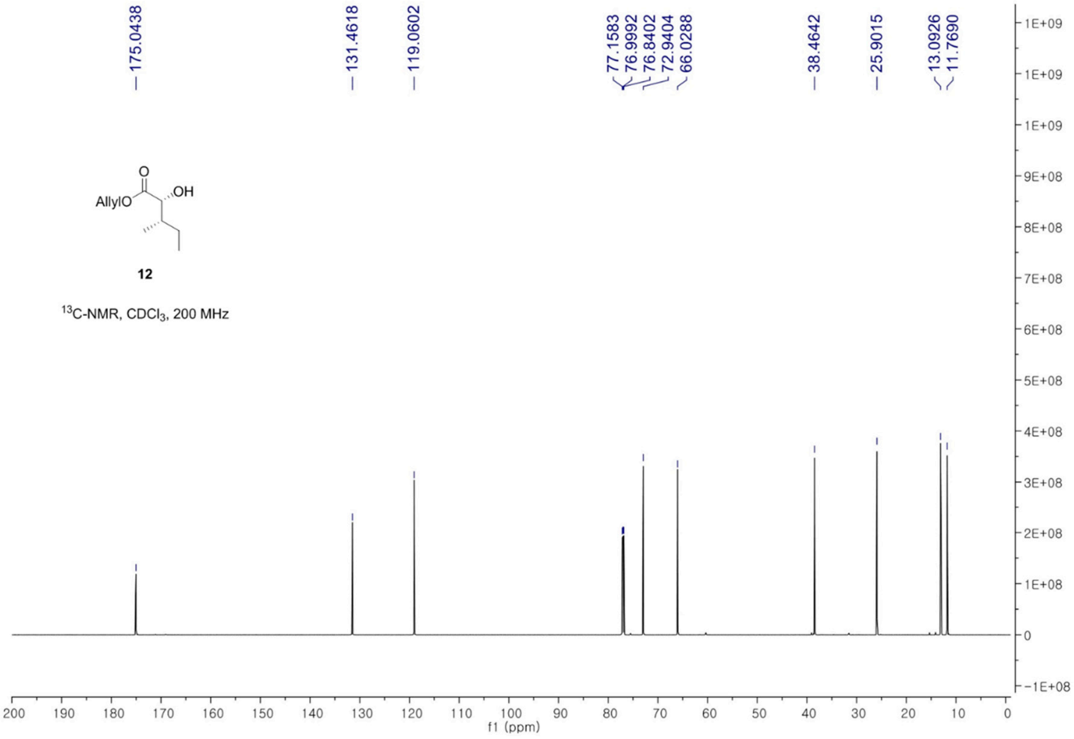

^1H- and ^{13}C-NMR Spectra of **13**

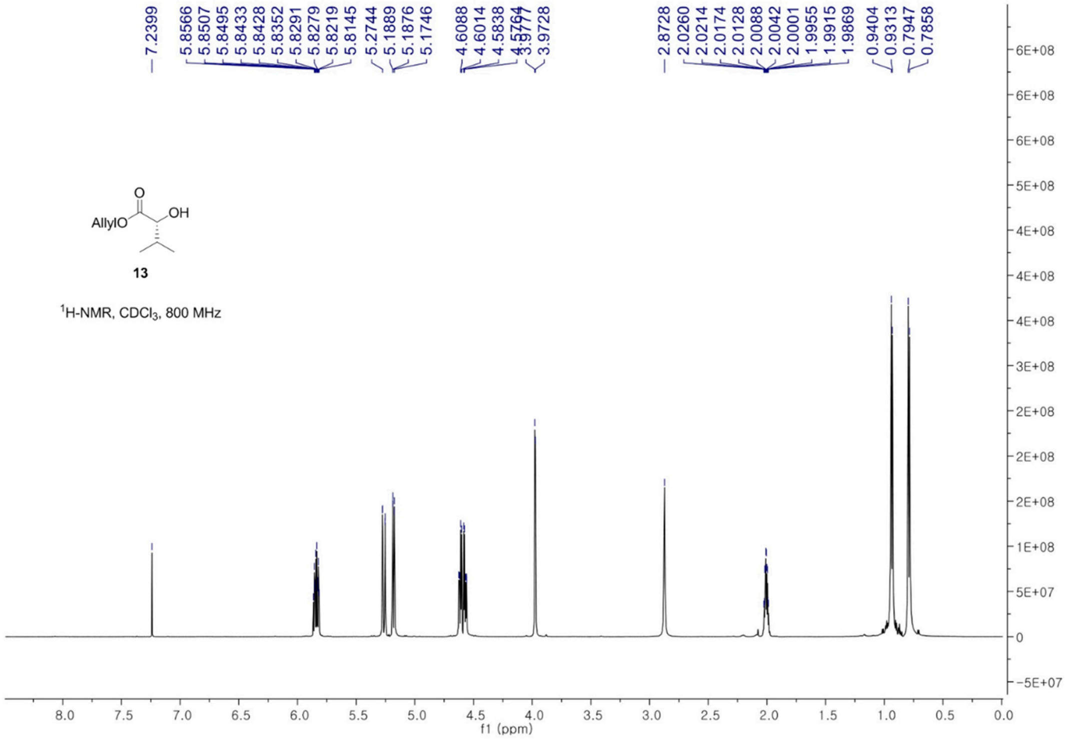

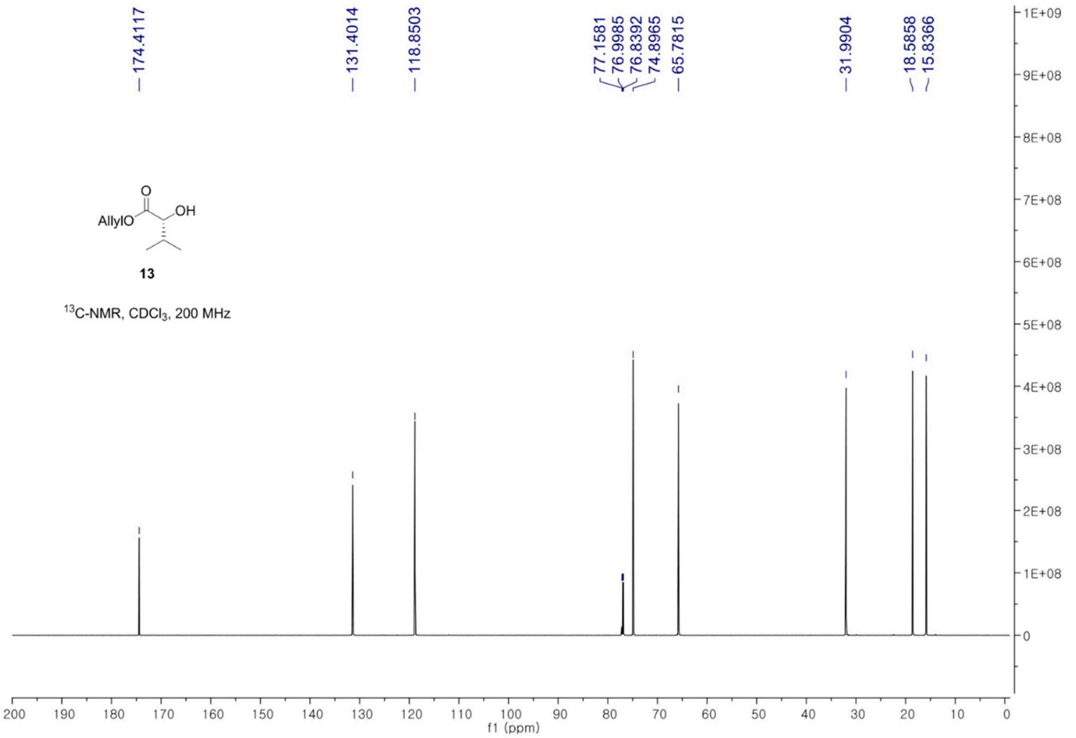

^{1}H- and ^{13}C-NMR Spectra of **35**

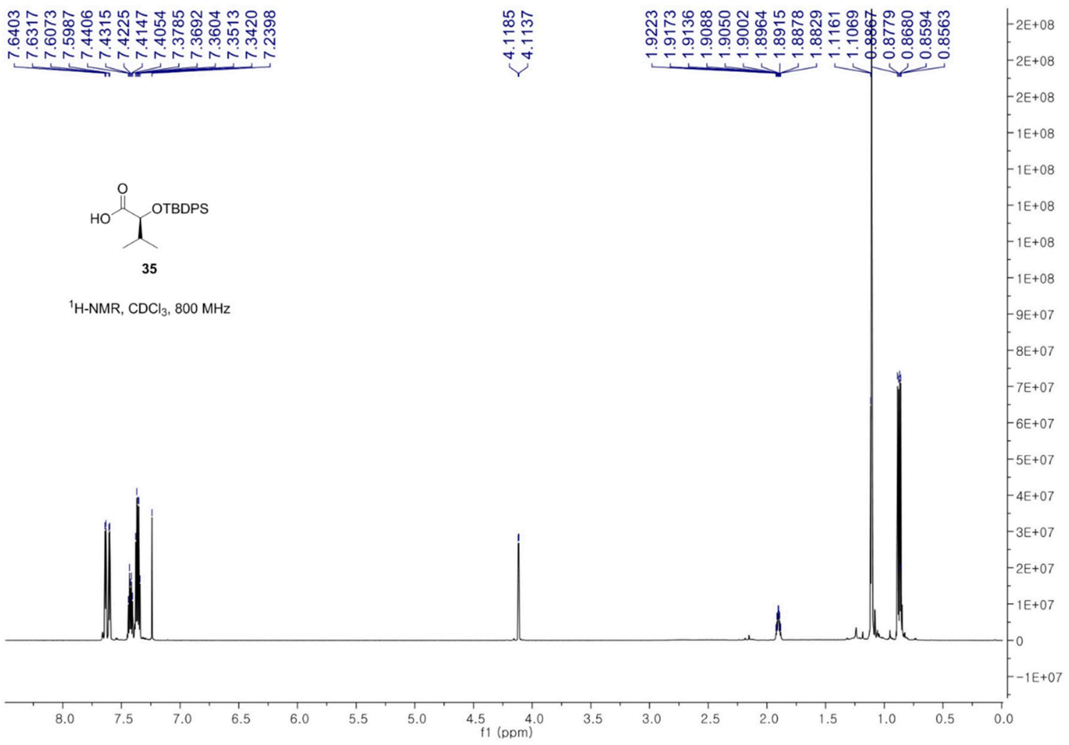

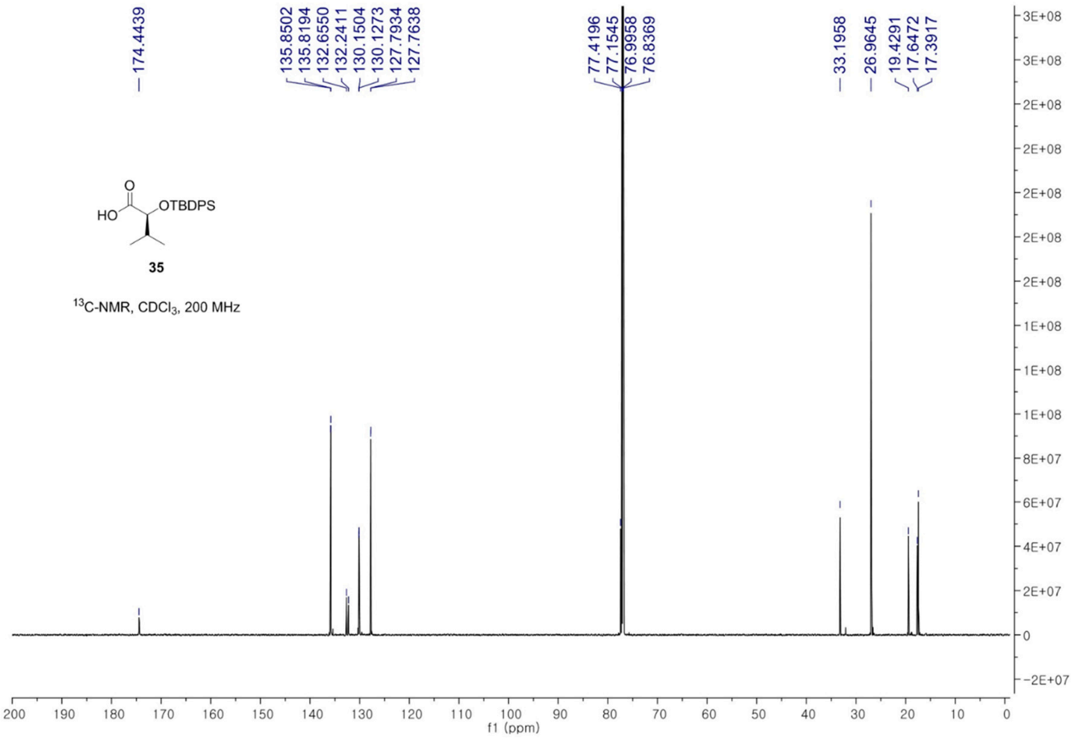

^{1}H- and ^{13}C-NMR Spectra of **36**

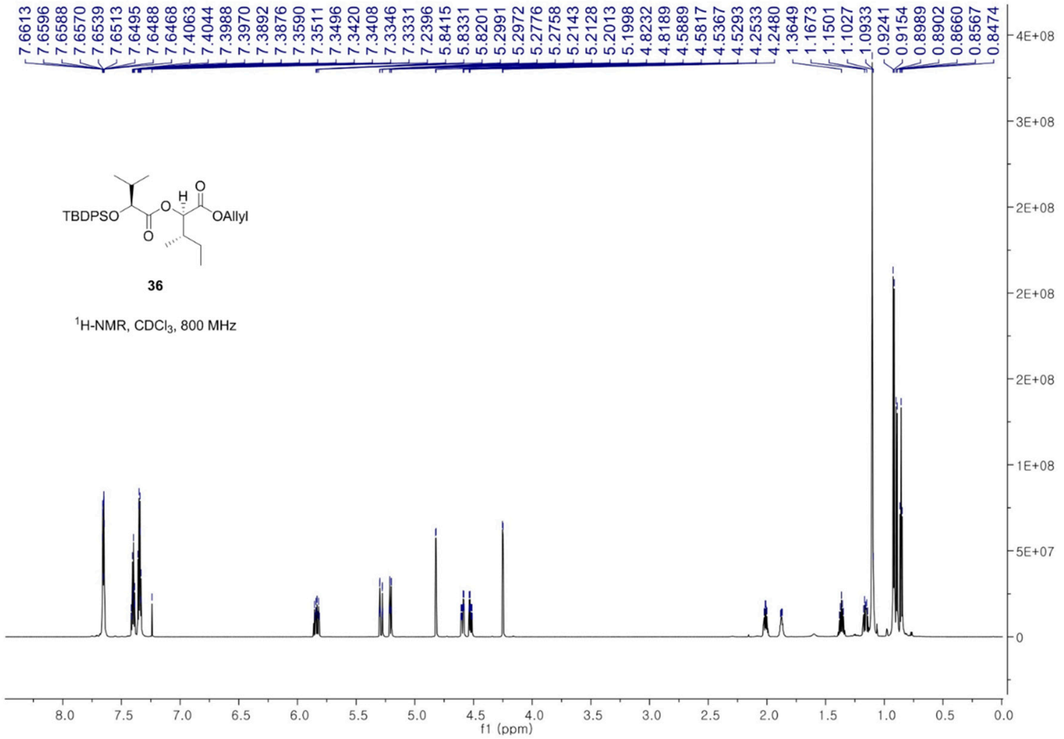

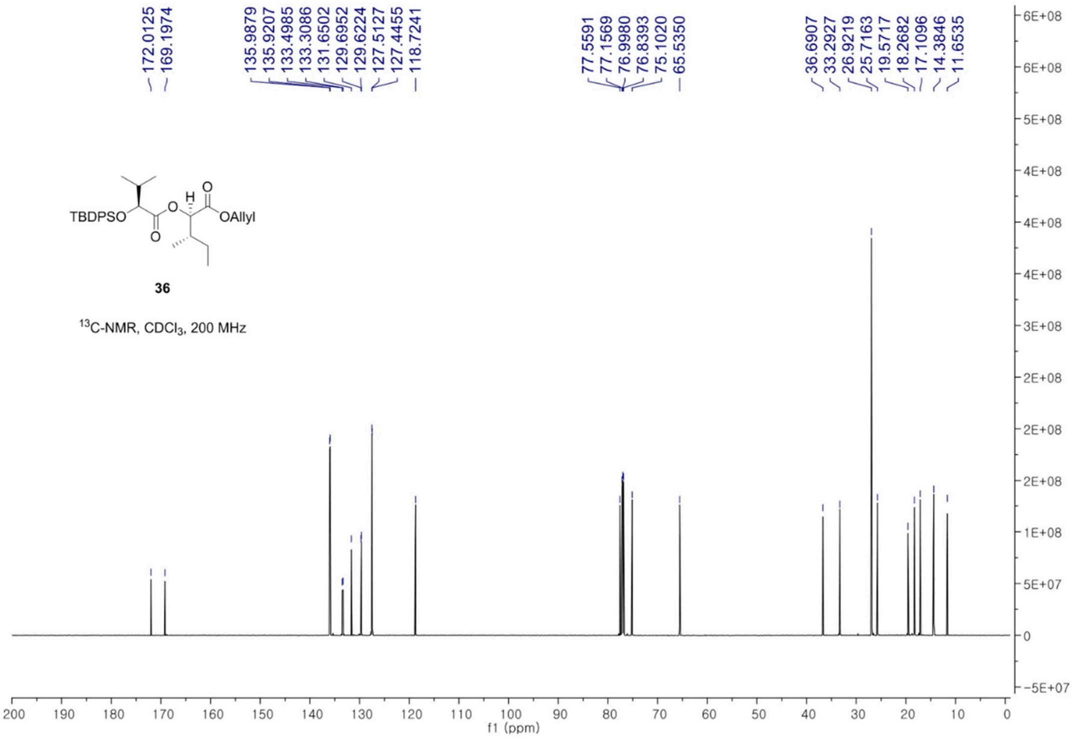

^{1}H- and ^{13}C-NMR Spectra of **37**

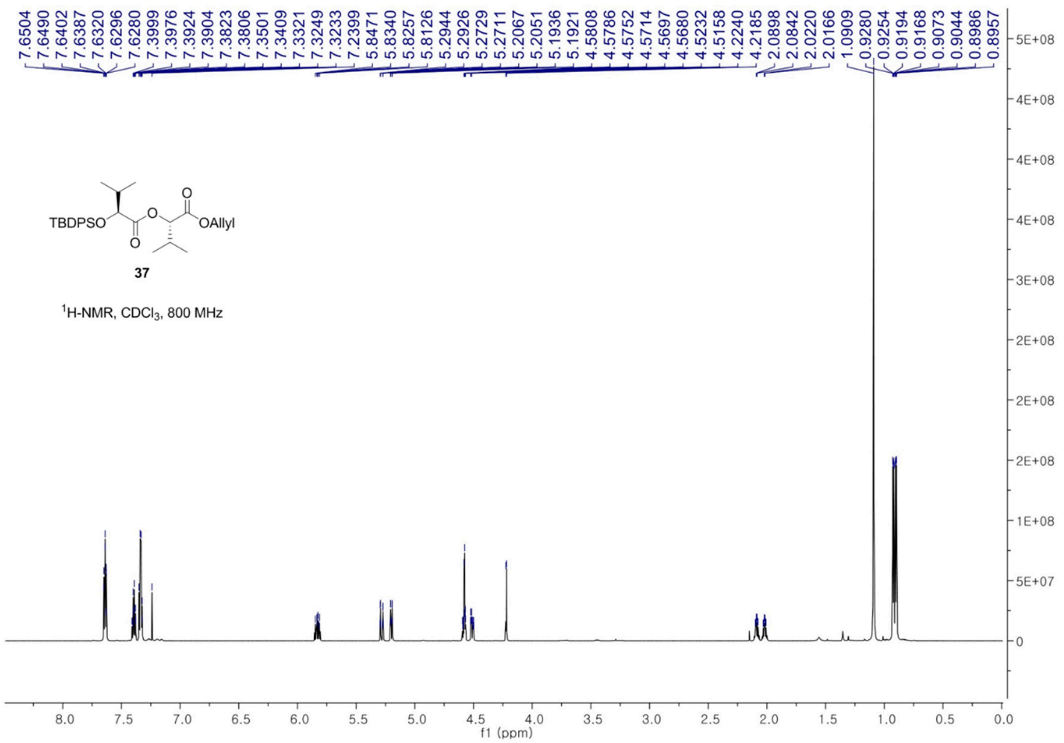

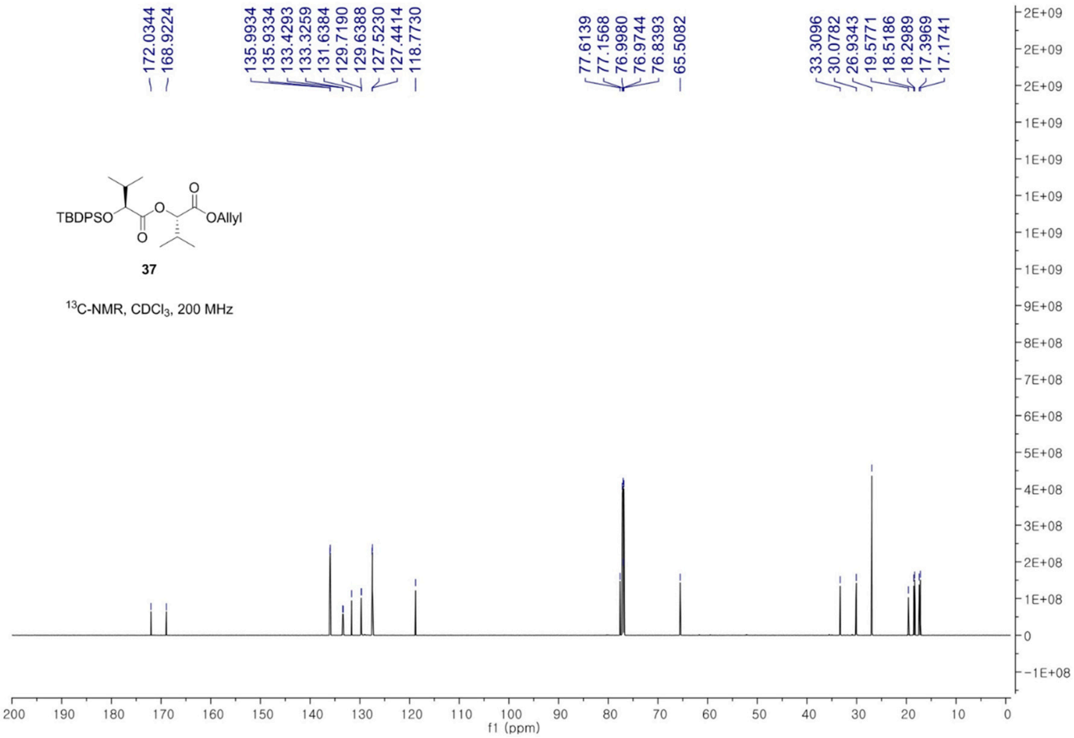

^{1}H- and ^{13}C-NMR Spectra of **38**

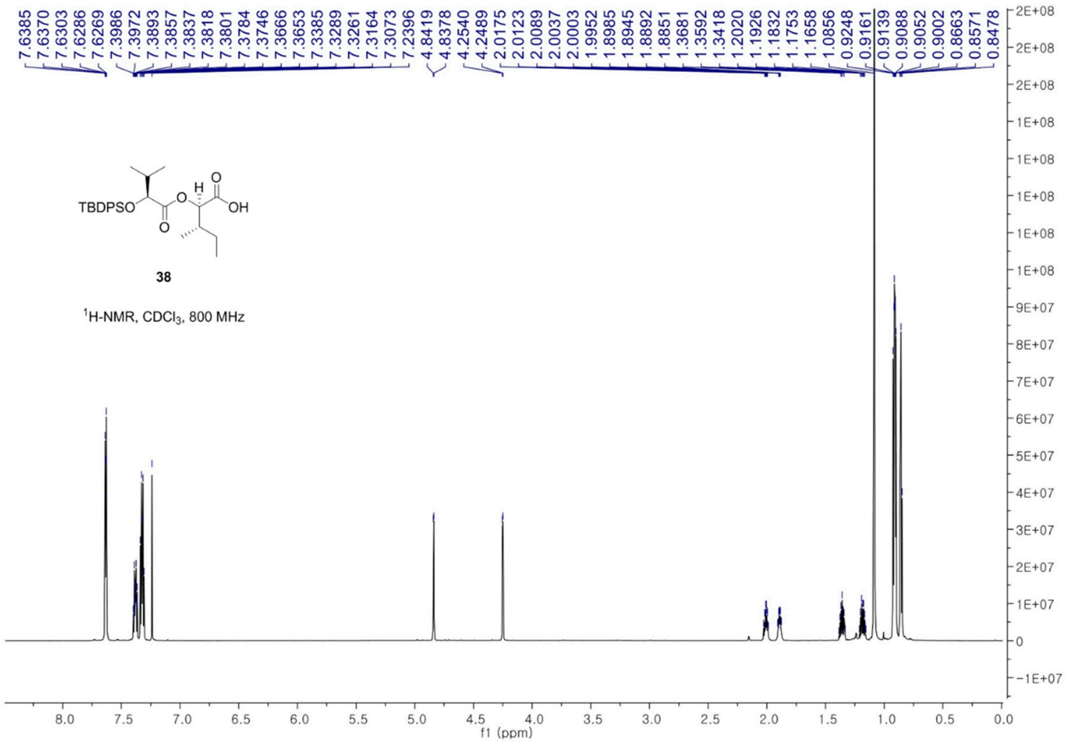

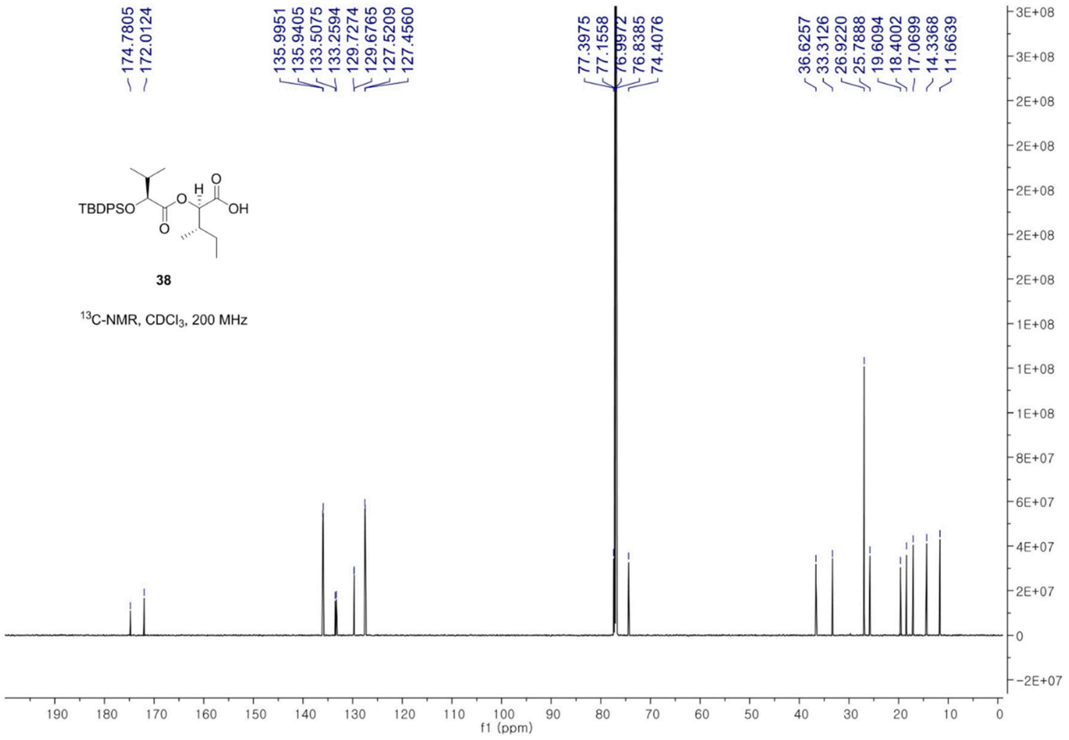

^{1}H- and ^{13}C-NMR Spectra of **39**

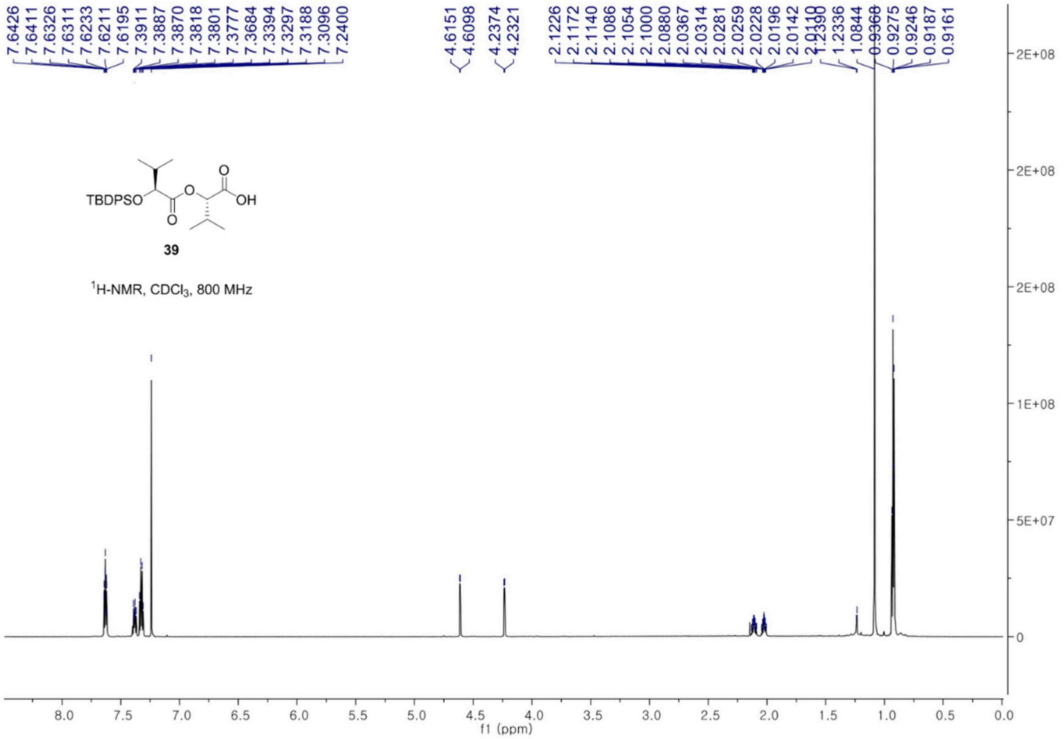

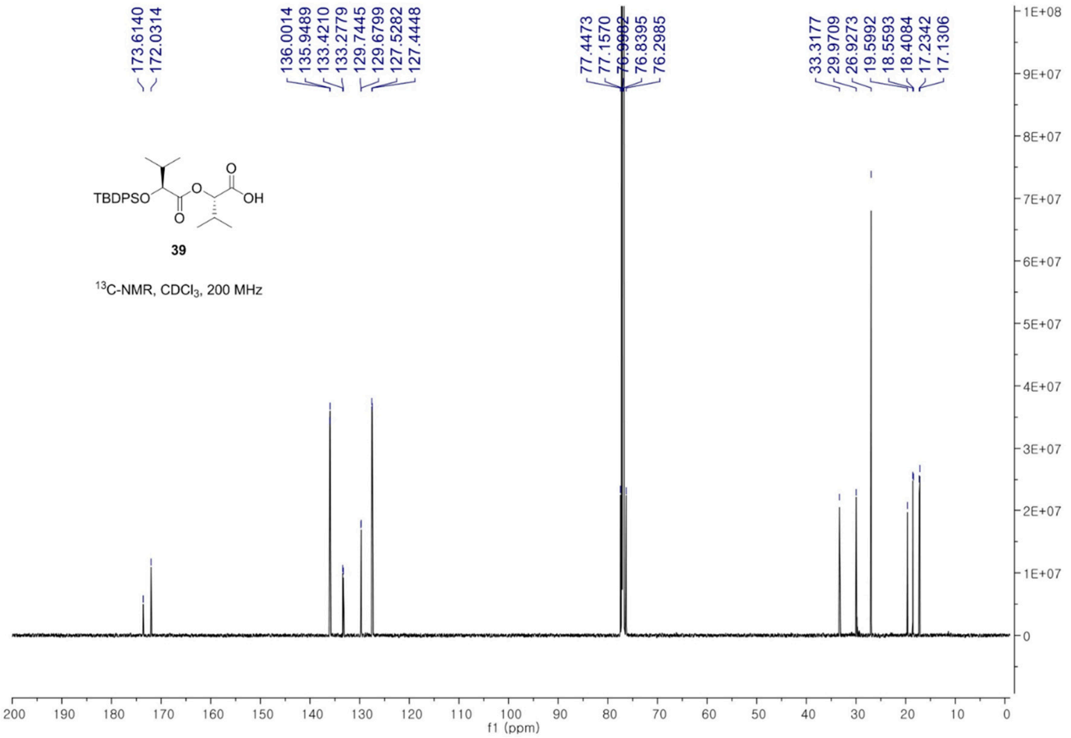

^{1}H- and ^{13}C-NMR Spectra of izenamide C (**3**)

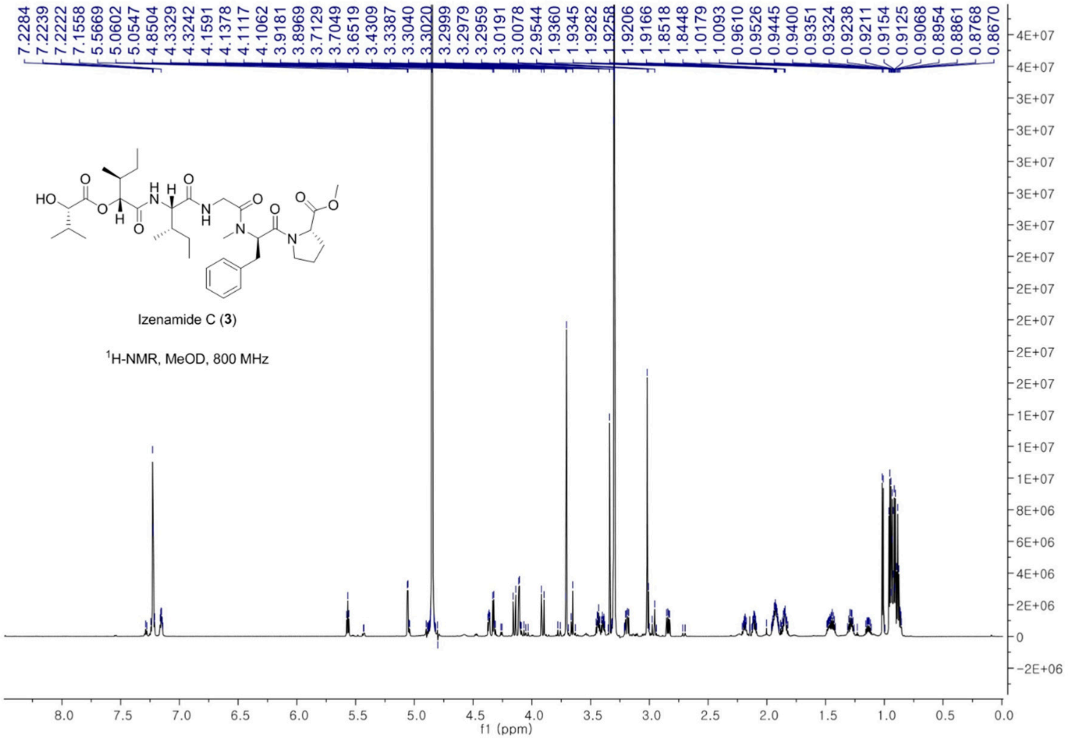

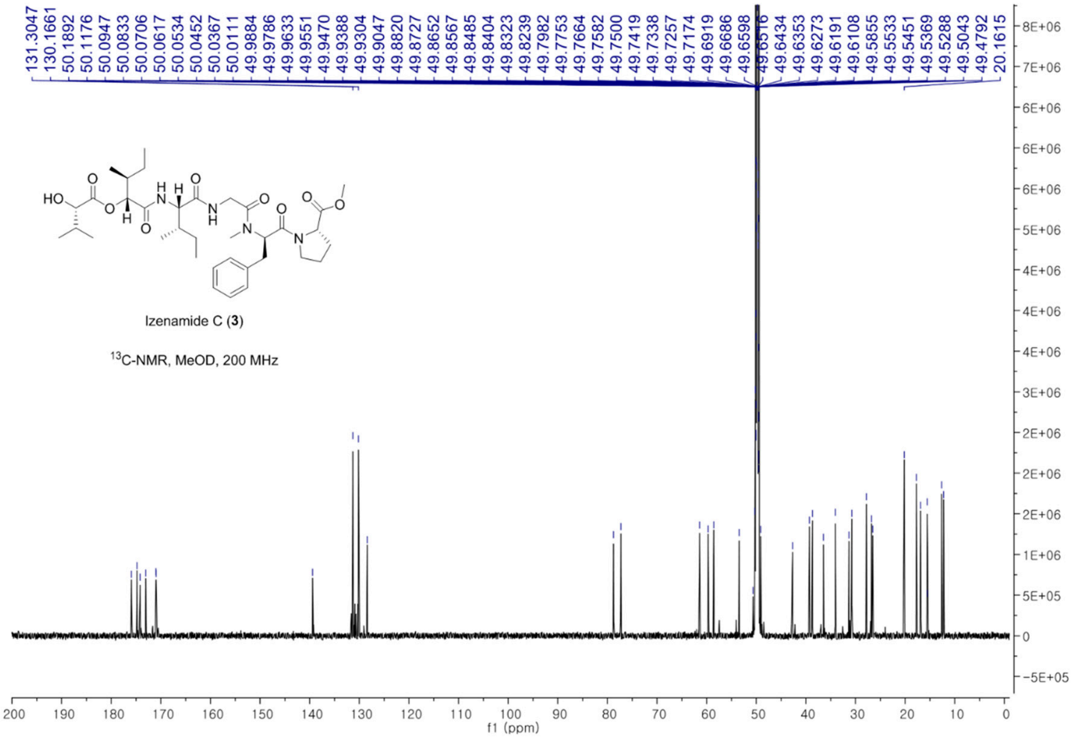

^{1}H- and ^{13}C-NMR Spectra of **42**

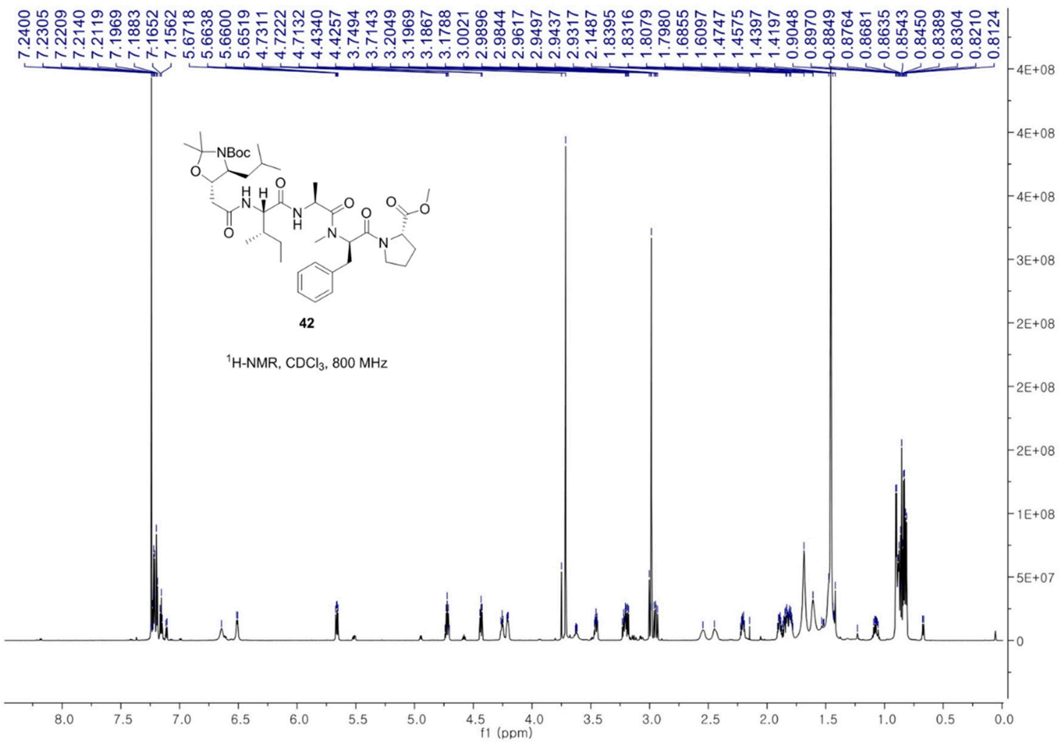

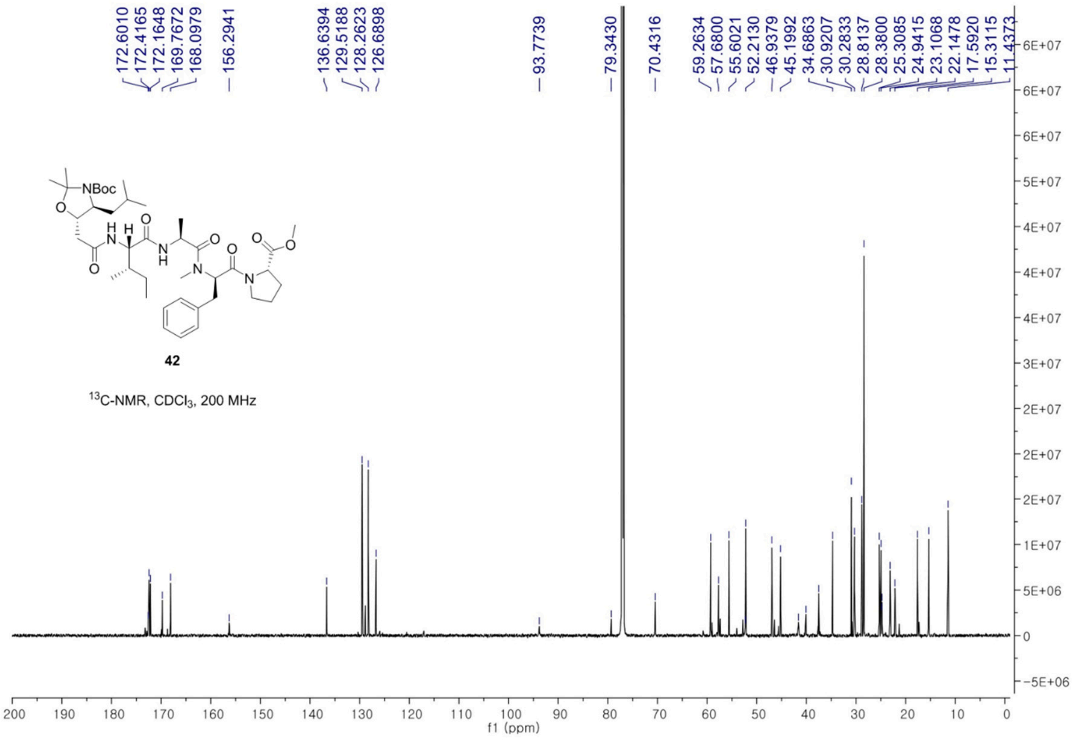

^{1}H- and ^{13}C-NMR Spectra of izenamide A (**1**)

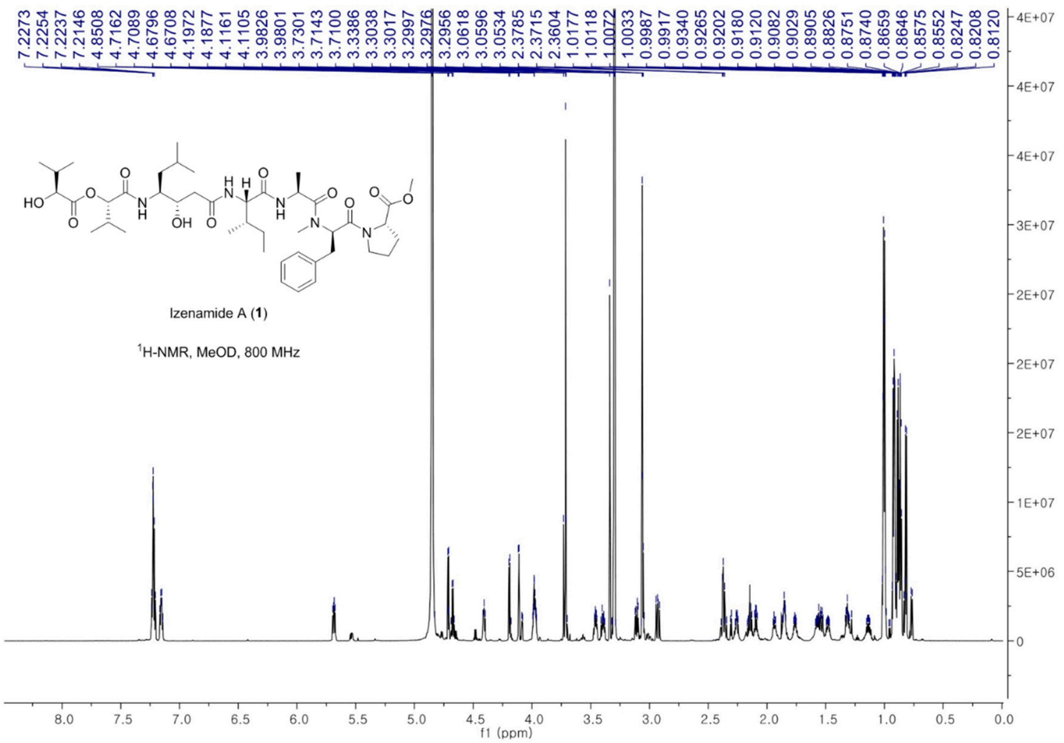

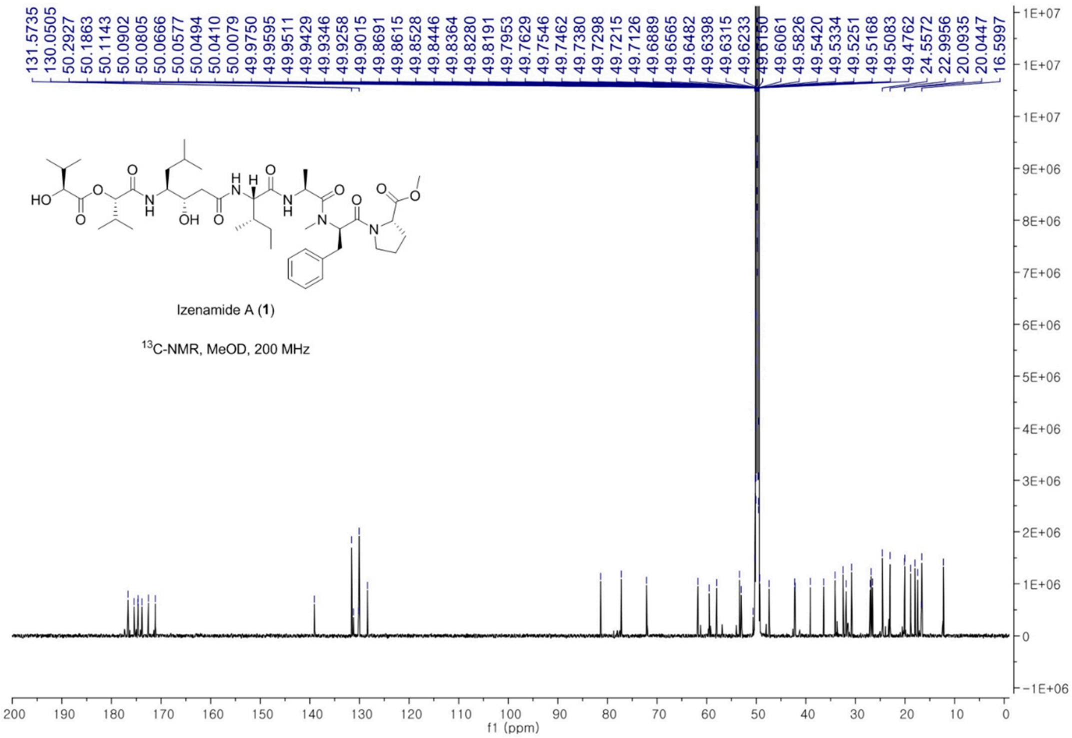

^{1}H- and ^{13}C-NMR Spectra of izenamide B (**2**)

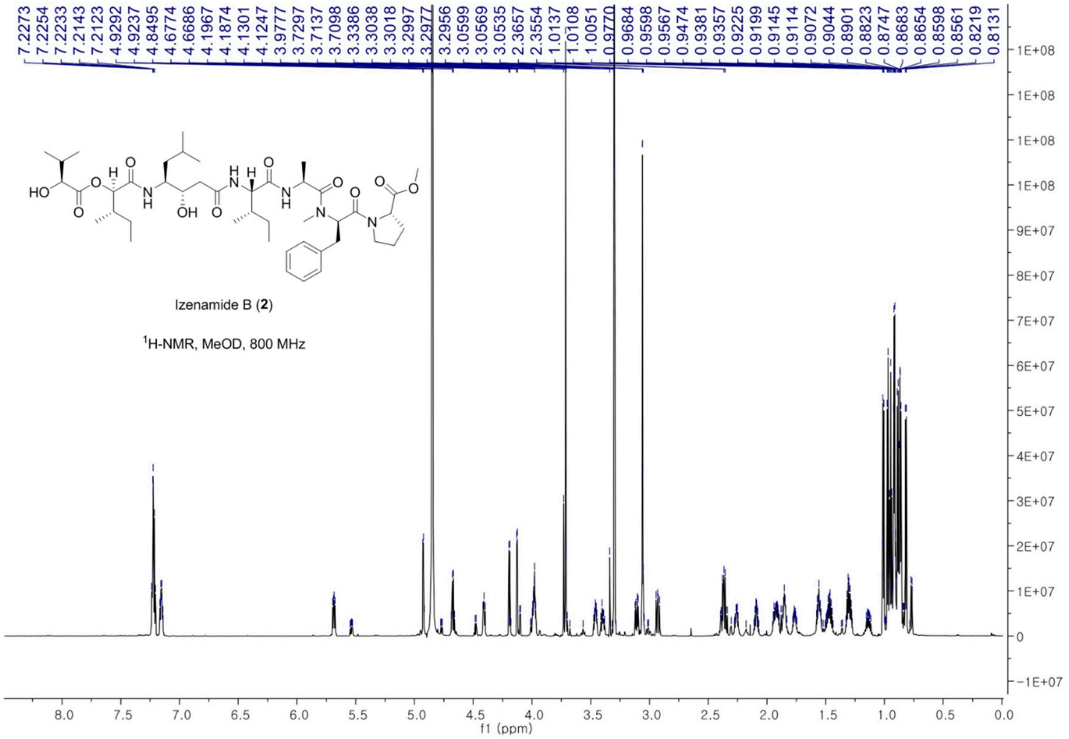

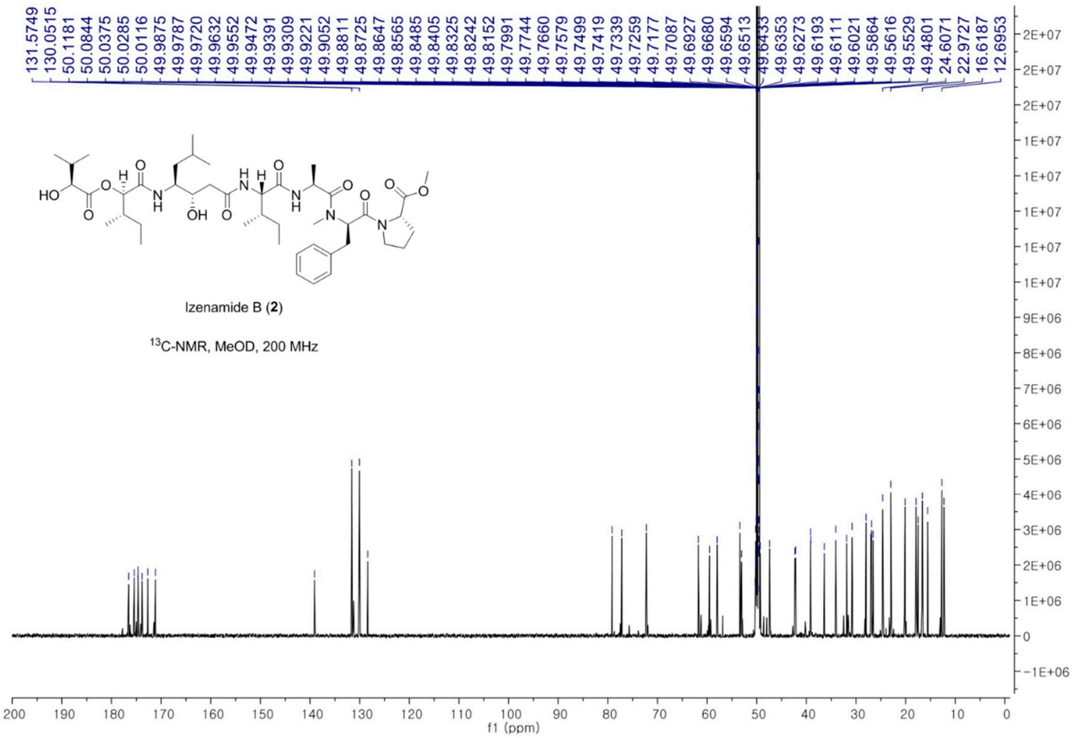

Supplement: Supplementary file 1 [file molecules-24-03424-s001.pdf]
